# Research Communication: Changing Aetiology of Chronic Liver Diseases in East Asia Pacific and HCC Surveillance in Non‐Cirrhotic Patients

**DOI:** 10.1111/apt.70428

**Published:** 2025-10-21

**Authors:** Ming Liu, Chuan Liu, Tsz Ngai Mok, Xiaolong Qi, Wai‐kit Ming

**Affiliations:** ^1^ Department of Infectious Diseases and Public Health Jockey Club College of Veterinary Medicine and Life Sciences, City University of Hong Kong Hong Kong China; ^2^ Liver Disease Center of Integrated Traditional Chinese and Western Medicine, Department of Radiology, Zhongda Hospital, Medical School Southeast University, Nurturing Center of Jiangsu Province for State Laboratory of AI Imaging & Interventional Radiology (Southeast University) Nanjing China; ^3^ Basic Medicine Research and Innovation Center of Ministry of Education, Zhongda Hospital, Southeast University; State Key Laboratory of Digital Medical Engineering Nanjing China; ^4^ Institute of Global Governance and Innovation for a Shared Future, City University of Hong Kong Hong Kong China

## Abstract

East Asia Pacific (EAP) faces a high burden of chronic liver disease (CLD), with metabolic dysfunction‐associated steatotic liver disease (MASLD) projected to account for about 80% of CLD prevalence by 2040. Using Global Burden of Disease 2021 data, we assessed CLD trends, transitions to hepatocellular carcinoma (HCC), and the cost‐effectiveness of non‐cirrhotic HCC surveillance. Despite declining viral hepatitis burden, MASLD and alcohol‐related CLD have risen. HCC surveillance in non‐cirrhotic patients was cost‐effective only for chronic hepatitis B in selected older age groups, with substantial variability across countries. These findings support refining surveillance guidelines and adaptation of risk stratification tools.

## Graphical abstract

East Asia Pacific faces a rising CLD burden, with MASLD projected to account for 80% of CLD prevalence by 2040. HCC surveillance in non‐cirrhotic patients is cost‐effective only in select older age groups. Wide variation in thresholds across countries highlights the need for updated guidelines and risk‐based surveillance strategies.

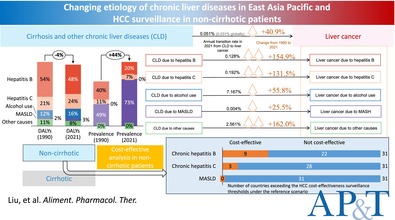

## 1 Introduction

East Asia and Pacific (EAP) bears a disproportionately high burden of liver cancer. Although hepatitis B virus (HBV) and hepatitis C virus (HCV) have historically driven the burden of chronic liver disease (CLD), the implementation of HBV vaccination programs and antiviral therapies has markedly reduced their impact. Concurrently, the prevalence of metabolic dysfunction‐associated steatotic liver disease (MASLD) and alcohol‐related liver disease has risen sharply, reshaping the etiological landscape [1, 2]. Hepatocellular carcinoma (HCC), which comprises 80% of primary liver cancers, remains highly prevalent in EAP and arises primarily in patients with CLD or cirrhosis, though approximately 20% of HCC cases occur in non‐cirrhotic individuals [3, 4].

While major liver society guidelines recommend HCC surveillance for cirrhotic patients, there is no consensus regarding non‐cirrhotic populations. Cost‐effectiveness thresholds for surveillance are well‐defined (e.g., > 0.2% annual incidence for HBV; > 1.5% for other etiologies), yet evidence supporting routine surveillance in non‐cirrhotic groups is limited [5]. Most recommendations for non‐cirrhotic HBV surveillance (e.g., Asian men over 40 and women over 50) are based on data from select East Asian cohorts, limiting generalizability [6]. This study aims to evaluate the evolving CLD burden and assess the cost‐effectiveness of HCC surveillance in non‐cirrhotic populations across EAP countries.

## 2 Methods

Full methodological details are provided in the Supporting Information.

## 3 Results

### 3.1 Burden of CLD in EAP

In 2021, EAP accounted for 35.8% of global prevalence and 27.4% of deaths. Prevalence rose by 43.8% from 1990 to 2021, while DALYs remained stable. ASPR slightly declined (EAPC: −0.50% [−0.63% to −0.37%]), with a sharper age‐standardised DALY rate (ASDR) decline (−2.42% [−2.48% to −2.36%]) (Tables S3–S5).

At the national level, hepatitis B was the leading cause of CLD‐related DALYs in 28 of 34 EAP countries and territories (82.3%), followed by hepatitis C in four countries (11.8%) and alcohol use in two (5.9%). Most countries (33 of 34) exhibited a downward trend in ASDR (Table S7).

### 3.2 Sex, SDI, Aetiology, and Age Group Stratification

**FIGURE 1:**
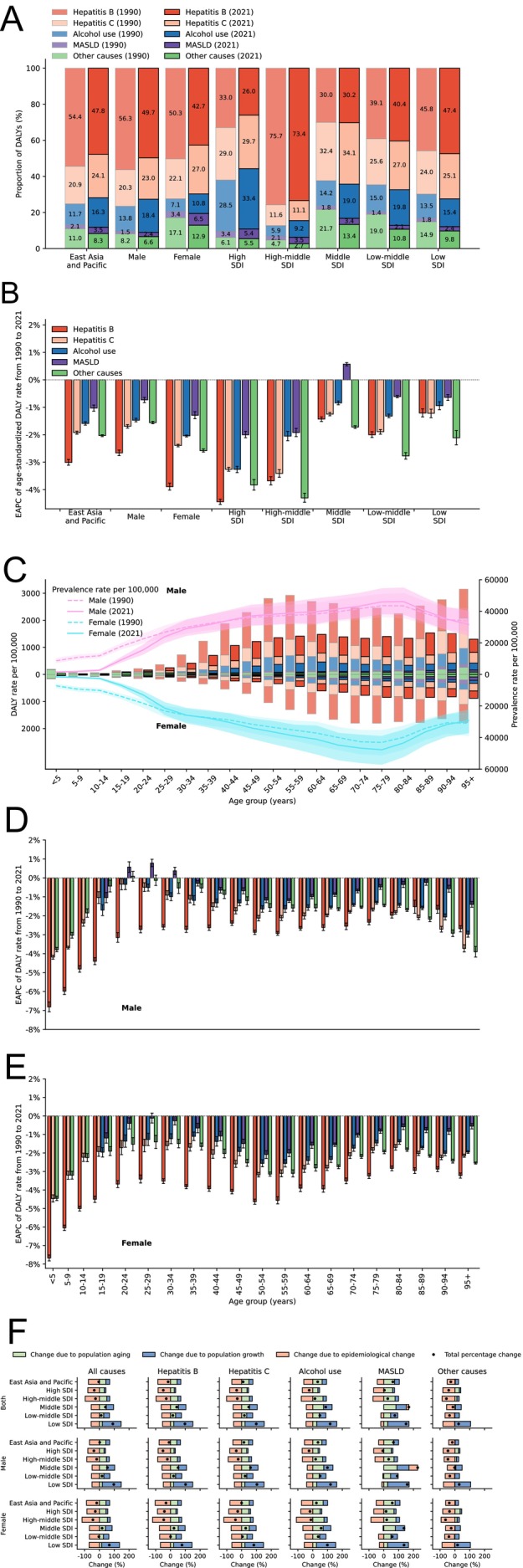
Etiological patterns, demographic disparities, and drivers of CLD burden in East Asia Pacific from 1990 to 2021. (A) Proportion of DALYs of CLD stratified by etiologies in EAP, across sexes and SDI regions in 1990 and 2021. (B) EAPC of ASDR of CLD stratified by etiologies in EAP, across sexes and SDI regions from 1990 to 2021. (C) DALY and prevalence rate of CLD stratified by etiologies in EAP, across sexes and age groups in 1990 and 2021. (D, E) EAPC of DALY rate of CLD stratified by etiologies in EAP across sexes and age groups from 1990 to 2021. (F) Decomposition of changes in DALYs of CLD stratified by etiologies in EAP from 1990 to 2021. The black circle represents the net change in DALYs for each aetiology. Bars illustrate the contribution of population growth, population aging, and epidemiological change to the total DALY change. Positive values (right of 0) indicate increases, while negative values (left of 0) reflect decreases. Colour‐coded sections highlight contributions across different time periods, summing to the overall change in DALYs. ASDR, age‐standardised disability‐adjusted life year rate; CLD, cirrhosis and other chronic liver diseases; DALYs, disability‐adjusted life years; EAP, East Asia Pacific; EAPC, estimated annual percentage change; MASLD, metabolic dysfunction–associated steatotic liver disease; SDI, socio‐demographic index.

In 2021, males (72.9%) were disproportionately affected. In males, hepatitis B (49.7%) and alcohol use (18.4%) were more prominent contributors. Middle and low‐middle SDI regions had higher ASDR in 2021. Hepatitis B (47.8%) remained the leading cause of DALYs, followed by hepatitis C (24.1%), while alcohol use (16.3%) and MASLD (3.5%) showed the most significant increases in their proportion of DALYs compared to 1990 (Figure 1A; Table S7).

Etiological burden varied across SDI regions, with alcohol use and MASLD more prominent in high SDI regions. Hepatitis B was predominant in high‐middle SDI regions (73.4%) and the leading cause in low‐middle (40.4%) and low (47.4%) SDI regions, while hepatitis C (34.1%) was the leading cause in middle SDI regions (Figure 1A).

The decline in ASDR in EAP was driven by the reduction in hepatitis B (EAPC: −3.01% [−3.11% to −2.90%]), followed by other causes, hepatitis C, alcohol use, and MASLD. These trends were consistent across sexes, with greater reductions observed in females. The most significant decrease was in high and high‐middle SDI regions, while low SDI regions saw significant DALY growth of 90.7% (Figure 1B; Figure S1D,H; Table S7).

The CLD burden was higher in individuals ≥ 40 years. DALYs from alcohol use were high in males aged ≥ 45 years, while MASLD affected males ≥ 85 years and females ≥ 75 years (Figure 1C–E).

### 3.3 Decomposition of Changes in DALYs

Population aging, population growth, and epidemiological change accounted for 44.0%, 25.4%, and −73.7% of DALYs change, respectively. Population growth primarily drove changes in low SDI regions, while aging had a more significant impact in high‐middle and middle SDI regions (Figure 1F).

### 3.4 Analysis of Risk Factors

In 2021, DALYs attributed to high alcohol use and drug use increased significantly across all SDI regions compared with those in 1990. This was particularly notable in low‐middle SDI regions (Figure S4).

### 3.5 Annual Transition Rates From CLD to Liver Cancer

From 1990 to 2021, incident cases and DALYs from liver cancer increased significantly while ASDR decreased by aetiology and sex. The incident cases and proportion of DALYs from alcohol use and MASH both increased in both sexes (Figure S6). The global annual transition rate from CLD to liver cancer increased by 28.7%, with EAP showing both a higher transition and a higher increase of 40.9%. The transition in EAP was higher in all etiologies than the global rate. Males, older age groups, and high SDI regions exhibited higher rates. High SDI regions led transitions due to hepatitis B, hepatitis C, and MASLD, while high‐middle SDI regions led in alcohol‐related transitions (Figure S7).

### 3.6 Correlations With SDI

A significant negative relationship was found between ASDR and SDI in high (ρ= −0.20, *p* = 0.005 < 0.01), middle (ρ= −0.49, *p* < 0.001), and low‐middle (ρ= −0.63, *p* < 0.001) SDI regions (Figure S8). Transition rates positively correlated with SDI in high (*ρ* = 0.60, *p* < 0.001) and low‐middle (*ρ* = 0.34, *p* < 0.001) SDI regions (Figure S11).

### 3.7 Predicted Trends in CLD and Liver Cancer

**FIGURE 2:**
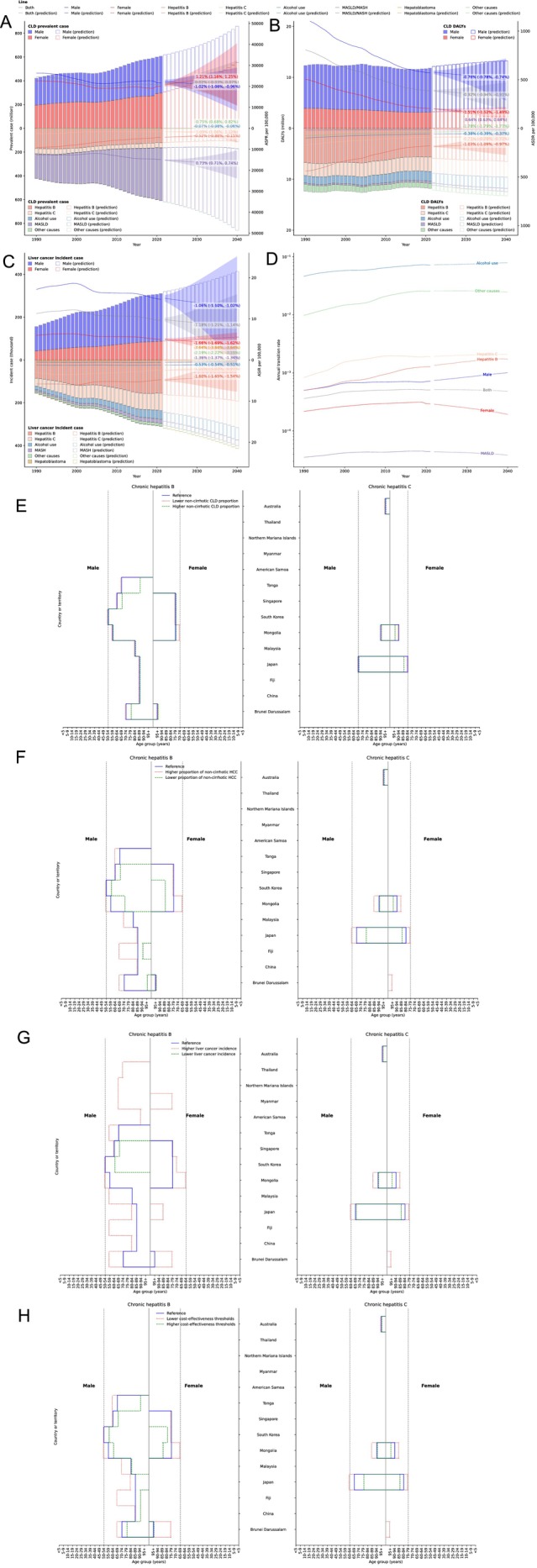
Projections to 2040 and cost‐effectiveness of hepatocellular carcinoma (HCC) surveillance in non‐cirrhotic populations in East Asia Pacific. (A) Number of prevalent cases and ASPR of CLD. (B) Number of DALYs and ASDR of CLD. (C) Number of incident cases and ASIR of liver cancer. (D) Annual transition rates from CLD to liver cancer. (E) Age groups and countries exceeding the cost‐effectiveness threshold for HCC surveillance in non‐cirrhotic chronic hepatitis B and hepatitis C under the reference scenario and scenarios assuming a lower and higher proportion of non‐cirrhotic chronic liver disease. (F) Age groups and countries exceeding the cost‐effectiveness threshold for HCC surveillance in non‐cirrhotic chronic hepatitis B and hepatitis C under the reference scenario and scenarios assuming a lower and higher proportion of non‐cirrhotic HCC cases. (G) Age groups and countries exceeding the cost‐effectiveness threshold for HCC surveillance in non‐cirrhotic chronic hepatitis B and hepatitis C under the reference scenario and scenarios assuming a lower and higher liver cancer incidence. (H) Age groups and countries exceeding the cost‐effectiveness threshold for HCC surveillance in non‐cirrhotic chronic hepatitis B and hepatitis C under the reference scenario and scenarios assuming lower and higher cost‐effectiveness thresholds. Projections were performed using the Integrated Nested Laplace Approximation (INLA) within a Bayesian age‐period‐cohort (BAPC) model framework. Population forecasts were derived from the United Nations World Population Prospects 2024 (medium fertility variant). ASDR, age‐standardised disability‐adjusted life year rate; ASPR, age‐standardised prevalence rate; CLD, cirrhosis and other chronic liver diseases; DALYs, disability‐adjusted life years; HCC, hepatocellular carcinoma; MASH, metabolic dysfunction–associated steatohepatitis; MASLD, metabolic dysfunction–associated steatotic liver disease. [Correction added on 29 October 2025, after first online publication: Figure 2G has been updated in this version.]

From 2022 to 2040, CLD prevalence is projected to increase. By 2040, MASLD is projected to contribute 79.95% of prevalence. In contrast, ASDR is predicted to decline overall, while MASLD‐related ASDR is anticipated to increase (0.64% [0.63% to 0.64%]). DALYs are expected to remain stable with hepatitis B and C continuing as the dominant contributors to DALYs. Liver cancer incident cases will rise, although ASIR will decline across sexes and etiologies (Figure 2A–D).

### 3.8 HCC Incidence in Non‐Cirrhotic Patients

The cost‐effectiveness threshold for initiating surveillance varies significantly by country. Under the reference scenario, nine countries (29.0%) exceeded the 0.2% surveillance threshold for hepatitis B, with cost‐effective surveillance identified for males aged ≥ 50 years in South Korea and females aged ≥ 75 years in three countries. For hepatitis C, three countries (9.7%) surpassed the 1.5% threshold. Notably, MASLD did not surpass the 1.5% threshold in any country or age group under any scenario. Sensitivity analyses yielded consistent results (Figure 2E,H; Tables S10–S18 in Excel).

## 4 Discussion

This study highlights the evolving burden of CLD in EAP and its implications for HCC surveillance in non‐cirrhotic populations. While the overall ASDR from CLD is declining, the absolute burden is rising due to population aging and the increasing prevalence of MASLD and alcohol use.

Intensive HBV vaccination programmes and antiviral therapies have significantly reshaped the landscape of liver diseases across the region. However, HBV prevalence remains higher in Asia compared to Western countries. Concurrently, rising alcohol consumption, likely associated with economic growth, has contributed to the growing burden of alcoholic liver disease (ALD), particularly in high‐SDI regions. Meanwhile, the prevalence of MASLD is increasing rapidly, fueled by sedentary lifestyles and overnutrition linked to rapid urbanisation. In many Asian countries, MASLD rates are now comparable to those in Western populations, especially among older adults [2].

While HCC surveillance in cirrhotic patients is well established, recommendations for non‐cirrhotic individuals vary widely across EAP countries. Some guidelines—such as those in China, Japan, South Korea, Taiwan, and Thailand—extend surveillance to non‐cirrhotic patients with chronic hepatitis B or C. Others, including Australia, Malaysia, the Philippines, and Singapore, limit recommendations to non‐cirrhotic hepatitis B carriers (Table S9). Surveillance was cost‐effective only for older adults with non‐cirrhotic hepatitis B in select countries, while routine surveillance for non‐cirrhotic hepatitis C and MASLD was generally not cost‐effective across any scenario or age group. These findings are consistent with American Association for the Study of Liver Diseases guidelines, which do not recommend routine surveillance in non‐cirrhotic MASLD or hepatitis C due to low HCC incidence [7]. Although MASLD‐related HCC frequently arises in non‐cirrhotic individuals, the incidence remains too low to justify universal screening. However, given the large at‐risk population, especially those with advanced fibrosis, individualized strategies using validated risk stratification tools (e.g., PAGE‐B, FIB‐4) may enable targeted and efficient surveillance [8]. For example, HCC surveillance is recommended for non‐cirrhotic chronic hepatitis B patients with a PAGE‐B score > 10 [8]. In primary care settings, patients with FIB‐4 < 1.3 and limited metabolic risk factors may be monitored less frequently, while those with diabetes or FIB‐4 ≥ 1.3 may require more intensive surveillance or referral [9]. A hybrid surveillance strategy may be optimal: universal surveillance could be justified in populations with high annual transition rates, while individual‐level risk tools can guide personalised screening decisions in lower‐risk groups.

Components of the metabolic syndrome, such as diabetes and obesity, are emerging factors for MASLD‐related HCC; however, further data and risk stratification tools are needed to identify non‐cirrhotic MASLD patients with sufficient risk to warrant HCC surveillance [8, 10].

This study has several limitations. Our analysis relies on GBD 2021 estimates, which may be affected by underreporting, regional data quality, and diagnostic inconsistencies. The absence of population‐based screening likely leads to underestimation of chronic liver disease and liver cancer, particularly among non‐cirrhotic individuals. MASLD diagnosis often depends on clinical, radiologic, or histologic criteria, and heterogeneous definitions may further reduce estimation accuracy [1, 10]. Screening for alcohol use disorder remains suboptimal. The burden of metabolic liver disease and ALD is likely underestimated [1, 3]. Multi‐aetiology splitting may oversimplify co‐existing risk factors and introduce misclassification. Despite these limitations, our findings provide important regional evidence to inform evolving HCC surveillance policies. Future research should focus on validating and implementing risk stratification tools to improve the precision and cost‐effectiveness of surveillance strategies.

## Author Contributions

**Ming Liu:** conceptualization, validation, investigation, methodology, software, data curation, formal analysis, writing – original draft, writing – review and editing, visualization, project administration. **Chuan Liu:** validation, data curation, writing – review and editing. **Tsz Ngai Mok:** data curation, validation, writing – review and editing, methodology. **Xiaolong Qi:** writing – review and editing, supervision, conceptualization, project administration, resources, methodology. **Wai‐kit Ming:** writing – review and editing, supervision, conceptualization, funding acquisition, project administration, resources, methodology, validation, investigation.

## Conflicts of Interest

The authors declare no conflicts of interest.

## Supporting information


**Data S1:** Supporting Information.

**Table S10:** Estimated annual incidence rate of HCC in non‐cirrhotic patients under the reference scenario.
**Table S11:** Estimated annual incidence rate of HCC in non‐cirrhotic patients assuming a lower proportion of non‐cirrhotic chronic liver disease.
**Table S12:** Estimated annual incidence rate of HCC in non‐cirrhotic patients assuming a higher proportion of non‐cirrhotic chronic liver disease.
**Table S13:** Estimated annual incidence rate of HCC in non‐cirrhotic patients assuming a lower proportion of non‐cirrhotic HCC cases.
**Table S14:** Estimated annual incidence rate of HCC in non‐cirrhotic patients assuming a higher proportion of non‐cirrhotic HCC cases.
**Table S15:** Estimated annual incidence rate of HCC in non‐cirrhotic patients assuming a lower liver cancer incidence.
**Table S16:** Estimated annual incidence rate of HCC in non‐cirrhotic patients assuming a higher liver cancer incidence.
**Table S17:** Estimated annual incidence rate of HCC in non‐cirrhotic patients assuming lower cost‐effectiveness thresholds.
**Table S18:** Estimated annual incidence rate of HCC in non‐cirrhotic patients assuming higher cost‐effectiveness thresholds.

## Data Availability

The data used in this study can be obtained from the GBD 2021 results page: https://vizhub.healthdata.org/gbd‐results/.
