# Supplementary material for: Research Communication: Changing Aetiology of Chronic Liver Diseases in East Asia Pacific and HCC Surveillance in Non‐Cirrhotic Patients
Source: Aliment Pharmacol Ther. 2025 Oct 21;63(4):569–73. doi: 10.1111/apt.70428 (PMC12854643; doi:10.1111/apt.70428)
Supplement: Supplementary file 1 — Data S1: Supporting Information. [file APT-63-569-s002.pdf]

Changing etiology of chronic liver diseases in East Asia Pacific and HCC surveillance in non-cirrhotic patients

Ming Liu, Chuan Liu, Tsz Ngai Mok, Xiaolong Qi, Wai-kit Ming

Table of Contents

Methods	Page 3-7
Supplementary Method 1: Decomposition analysis	Page 7-8
Table S1. ICD codes used in CLD estimation	Page 9
Table S2a. GBD locations of countries and territories in the East Asia Pacific region defined by the World Bank	Page 9-10
Table S2b. GBD 2021 quintile cutoffs	Page 10
Table S2c. Corresponding rules for calculation of annual transition rate from CLD to liver cancer	Page 10
Table S2d. Proportion of HCC in non-cirrhotic patients	Page 10-11
Table S3. Prevalent cases and ASPR of CLD in 1990 and 2021, and temporal trend from 1990 to 2021	Page 12-14
Table S4. DALYs and ASDR of CLD in 1990 and 2021, and temporal trend from 1990 to 2021	Page 15-17
Table S5. Death cases and ASMR of CLD in 1990 and 2021, and temporal trend from 1990 to 2021	Page 18-20
Table S6. Prevalent cases and ASPR of CLD in EAP in 1990 and 2021, and temporal trend from 1990 to 2021	Page 21-26
Table S7. DALYs and ASDR of CLD in EAP in 1990 and 2021, and temporal trend from 1990 to 2021	Page 27-32
Table S8. Death cases and ASMR of CLD in EAP in 1990 and 2021, and temporal trend from 1990 to 2021	Page 33-38
Table S9. Current HCC surveillance recommendations for cirrhotic and non-cirrhotic patients in EAP	Page 39-40
Fig. S1. Changes in prevalence and DALYs of CLD in EAP	Page 41-42
Fig. S2. EAPC of ASDR of CLD stratified by etiologies in EAP, across genders and countries or territories from 1990 to 2021	Page 43
Fig. S3. ASDR of CLD stratified by etiologies in EAP, across genders and countries or territories in 2021	Page 44
Fig. S4. Proportion of DALYs for CLD by risk factors, stratified by etiology, across genders and countries or territories in EAP in 1990 and 2021	Page 45
Fig. S5. Proportion of DALYs for CLD by risk factors, stratified by etiology, across genders and countries or territories in EAP in 1990 and 2021	Page 46

Fig. S6. Changes in incidence and DALYs of liver cancer in EAP Page 47
Fig. S7. Annual transition rate from CLD to liver cancer in EAP by sexes, age groups, SDI regions, and etiologies Page 48-49
Fig. S8. Relationship between ASDR of CLD and SDI by SDI regions and etiologies in EAP from 1990 to 2021 Page 49-50
Fig. S9. Annual transition rates from CLD to liver cancer in EAP, stratified by country and etiology, in 2021 Page 50-51

Fig. S10. EAPC of annual transition rates from CLD to liver cancer in EAP, stratified by country and etiology, from 1990 to 2021 Page 52-53

Fig. S11. Relationship between annual transition rates and SDI, stratified by SDI region and etiology, in EAP from 1990 to 2021 Page 53-54

Methods

GBD 2021 estimation framework

This study utilized data from the Global Burden of Disease (GBD) 2021 study coordinated by the Institute for Health Metrics and Evaluation. The GBD 2021 study provides an updated, comprehensive estimation of the disease burden, injuries, and risk factors for 204 countries and territories.[1] CLD is defined to include both cirrhosis and non-cirrhotic liver conditions with the potential to progress to cirrhosis. CLD is categorized into five etiologies: hepatitis B, hepatitis C, alcohol use, and other causes. These categories encompass both cirrhotic and non-cirrhotic stages, including non-cirrhotic MASLD, chronic hepatitis B, and chronic hepatitis C.[2] Similarly, liver cancer is further stratified into six etiologies: liver cancer due to hepatitis B, liver cancer due to hepatitis C, liver cancer due to alcohol use, liver cancer due to metabolic dysfunction–associated steatohepatitis (MASH), hepatoblastoma, and liver cancer due to other causes. We obtained annual estimates for incidence, prevalence, deaths, and DALYs for CLD and related risk factors, as well as liver cancer incidence, from the GBD 2021 Results Tool.[3]

The incidence and prevalence of cirrhosis were modeled using hospital discharge data and claims data from the Disease Modelling Meta-Regression (version 2.1) (DisMod-MR 2.1). To determine the etiologic distribution of cirrhosis, data from case series reporting the proportion of cases attributable to alcohol, hepatitis B, hepatitis C, MASLD, and other causes were used. In instances where patients had multiple etiologies, these multi-etiology cases were reassigned to a single etiology prior to modeling, based on intra-study proportional redistribution. For example, if a study reported 100 cirrhosis cases, 40 due to hepatitis B, 20 to alcohol, 30 to MASLD, and 10 to both hepatitis B and alcohol, the 10 overlapping cases would be proportionally redistributed between hepatitis B and alcohol, without altering the MASLD count. This approach was also applied to liver cancer data involving multiple etiologies. Five single-parameter models of five etiological proportions were developed using DisMod-MR 2.1.[4]

The GBD 2021 cause-of-death database for cirrhosis included data sources such as 23,117 vital registrations, 1,251 verbal autopsies, and five minimally invasive tissue samples.[1] Data processing corrections were applied to standardize these data for comparison by cause, age, sex, location, and time. Deaths without sufficient age data underwent age and sex splitting to assign GBD age and sex groups. Garbage codes (non-specific, implausible, or intermediate cause of death codes) were redistributed to appropriate targets to assign the underlying cause of death, International Classification of Diseases (ICD) codes for cirrhosis mapped to GBD for ICD-9 and ICD-10 (Table

S1).[5] Data sources with more than 50% of all deaths assigned to major garbage codes were adjusted, and a buffer system ensured consistency in data source inclusion between GBD cycles. Total cirrhosis mortality was modeled using the standard Cause of Death Ensemble modelling approach, and proportions of cirrhosis due to each aetiology were modeled using DisMod-MR 2.1.[4] Years of life lost (YLLs) were computed by multiplying the number of deaths for each cause-age-sex-location-year by the standard life expectancy at each age.[1] The liver cancer estimation followed the general framework for the GBD 2021 cancer estimation. Cancer incidence is directly estimated from cancer mortality estimates using mortality-to-incidence ratios (MIRs).[4]

DALYs were computed by summing the YLLs and years lived with disability (YLDs). YLDs were calculated by multiplying prevalence by disability weight, derived from community surveys and an open Internet survey.[4] GBD 2021 metrics were estimated as counts, all-ages and age-specific rates per 100,000 population, and age-standardized rates per 100,000 population, using the GBD standard population structure. The SDI is a composite indicator of social and economic conditions influencing health outcomes, calculated as the geometric mean of indices for the total fertility rate under 25 years old, mean education for those 15 years old and older, and lag-distributed income per capita. Each GBD location was assigned to an SDI region (high, high-middle, middle, low-middle, and low) based on its SDI value for 2021.[4]

Data analysis

Calculation of age-standardized rates of SDI regions in EAP

EAP countries and territories were mapped into GBD locations and grouped into SDI regions according to GBD 2021 quintiles (Table S2a, S2b) [6]. Since there are no predefined SDI regions for EAP, we calculated each SDI region's age-standardized rate (ASR) using the population and respective value in each country within the specific SDI region. The ASR for each SDI region group (g) was calculated using the following equation.

$$ASR_{g,j} = \frac{\sum_{i\in g}(ASR_{i,j} \times Pop_{i,j})}{\sum_{i\in g} Pop_{i,j}}$$

where the population of each country i in year j, was denoted as $Pop_{i,j}$.

Trend analysis of age-standardized rates

We analyzed the trends of ASR by calculating their respective estimated annual percentage change (EAPC). EAPC is an indicator for assessing the ASR trend over a time interval. We applied a regression model to the natural logarithm of rates using the equation. $y = \alpha + \beta x + \epsilon$, where y represents $\ln(ASR)$ and x represents the calendar year. EAPC was calculated as $100 \times (\exp(\beta) - 1)$ along with its 95% CI.

Positive EAPC estimates and the lower boundary of their 95% CI greater than zero indicate an increasing trend in the ASR. Conversely, if both the EAPC estimate and the upper boundary of the 95% CI are less than zero, the ASR is considered to have a decreasing trend. Stable trends are identified when the EAPC estimate is not statistically significant or the 95% CI spans zero.[7, 8]

Decomposition analysis of DALYs changes and risk factor analysis

To analyze the drivers of change in DALYs, we decomposed the changes in DALYs from 1990 to 2021 into three explanatory components: population growth, population aging, and epidemiological change. We refer to all changes in age-specific, sex-specific, and cause-specific DALY rates not explained by demographic change (population growth and aging) as epidemiological change. The decomposition analysis uses methods developed in demographic research by Das Gupta.[9] The detailed method is shown in Supplementary Method 1. Additionally, we analyzed the attributable burden of CLD due to risk factors such as high alcohol use and drug use. High alcohol use and drug use were defined according to the risk factor definitions used in GBD 2021. High alcohol use is defined as alcohol consumption in excess of the theoretical minimum risk exposure level, the level of alcohol consumption at which all-cause risk is minimized. Drug use includes the consumption of illegal drugs, the misuse of prescription medications, and the use of substances in a manner that is harmful to health.[10] The proportion of DALYs attributable to these factors was examined across sexes, SDI regions, etiologies, and countries.

Estimation of annual transition rate from CLD to liver cancer

The annual transition rate can be calculated using the following formula:

$$Annual\ transition\ rate = \frac{Inc_{i,y,e}}{Prev_{i,y,e}}$$

Where $Inc_{i,y,e}$ is the incident liver cancer cases in the age group i, year y, attributable to etiology e; $Prev_{i,y,e}$ is the prevalent CLD case in age group i, year y, attributable to etiology e.

This rate represented the annual probability of progression from CLD to liver cancer within a defined age group and etiology. The calculation is stratified by age, year, and etiology to account for variations in disease progression across demographics and over time. The corresponding relationships between etiologies in CLD and liver cancer are detailed in Supplementary Table S2c.

Relationship between ASDR of CLD and SDI, and annual transition rate and SDI

Furthermore, to assess the relationship between ASDR of CLD and SDI, and annual

transition rates and SDI across SDI regions and etiologies, we calculated Spearman's rank correlation coefficients (ρ) to assess the strength and direction of the monotonic relationship. Spearman's correlation is particularly suitable for capturing non-linear but monotonic relationships.[11] The statistical significance of the correlation was evaluated using P-values. A P-value less than 0.05 is considered statistically significant. Generalized Additive Models (GAM) were used to fit the relationships using spline functions. The GAM-fitted lines, along with the 95% CI, were plotted.

Prediction of trends in CLD and liver cancer

We projected future trends in prevalence, DALYs, age-standardized prevalence rates (ASPR), ASDR of CLD, and incidence and age-standard incidence rates (ASIR) of liver cancer in EAP from 2022 to 2040. Additionally, we predicted the annual transition rates from CLD to liver cancer. Predictions were made using the Integrated Nested Laplace Approximation (INLA) framework combined with the Bayesian age-period-cohort (BAPC) model. The BAPC model was fitted using a Poisson model with INLA used to project disease rates.[12, 13] Population projections were obtained from the World Population Prospects 2024 by the United Nations, using the medium fertility variant scenario.[14]

Estimation of annual HCC incidence in non-cirrhotic CLD patients in 2021

To estimate the annual incidence of HCC in non-cirrhotic patients, including chronic hepatitis B, C, and MASLD for 2021, we first derived the proportion of cirrhosis within total CLD prevalence for each etiology based on GBD 2017 estimates for decompensated and compensated cirrhosis.[15, 16]

The proportion of HCC cases arising in non-cirrhotic patients was obtained through a random-effects meta-analysis of 15 EAP cohort studies (Table S2d), Using Stata's metaprop command, stratified by etiology, the pooled proportions were: hepatitis B: 27.6% (95% CI: 20.8% to 34.8%; k=11; I^2=97.1%); hepatitis C: 28.4% (95% CI: 18.3% to 39.7%; k=5; I^2=90.0%); MASLD: 41.2% (95% CI: 28.9% to 54.1%; k=13; I^2=92.4%).

We assumed that HCC constitutes 80% of all liver cancer cases.[17] The annual incidence of HCC in non-cirrhotic patients was calculated using the following formula:

$$Annual\ HCC\ incidence = \frac{Inc_{2021,e} \times 80\%}{Prev_{2021,e} \times (1 - Pro_{cirrhosis,e})}$$

Where $Inc_{2021,e}$ is the incident liver cancer cases in 2021 attributable to etiology e;

$Prev_{2021,e}$ is the prevalent CLD case in 2021, attributable to etiology e;
$Pro_{cirrhosis}$ is the proportion of cirrhosis, attributable to etiology e.

Cost-effectiveness thresholds were set at 1.5% for non-cirrhotic chronic hepatitis C and MASLD and 0.2% for non-cirrhotic chronic hepatitis B, based on established guidelines.[18, 19]

Sensitivity analyses were performed to evaluate robustness:
1.Non-cirrhotic CLD proportion: scenarios assuming a 10% higher or 10% lower than the non-cirrhotic CLD proportion in 2017, limited up to 100%
2.Proportion of non-cirrhotic HCC: scenarios using the upper and lower 95% CI bounds from pooled meta-analyses.
3.Liver cancer incidence: scenarios using the upper and lower 95% uncertainty intervals (UI).
4.Cost-effectiveness thresholds: scenarios applying alternative thresholds—HBV: 0.15% and 0.25%; HCV and MASLD: 1.0% and 2.0%.
All statistical analyses were conducted using R (version 4.2.3) and Stata (vesion 17.0), and plotting was performed using Python's Matplotlib package (version 3.9.1).
Supplementary Method 1
Decomposition analysis
The decomposition methodology of Das Gupta[9, 20, 21] was introduced. The DALYs were decomposed by age structure, population growth, and epidemiologic changes. We refer to all changes in age-specific, sex-specific, and cause-specific DALY rates not explained by demographic change (population growth and aging) as epidemiological change. The age structure was described as the proportion of the population in the 20 age groups, including <5 years,5-9 years,10-14 years,15-19 years,20-24 years,25-29 years,30-34 years,35-39 years,40-44 years,45-49 years,50-54 years,55-59 years,60-64 years,65-69 years,70-74 years,75-79 years,80-84 years,85-89 years,90-94 years and 95+ years, defined in GBD 2021. The number of DALYs can be obtained from:

$$DALY_{a_y,p_y,r_y} = \sum_{i=1}^{20} a_{i,y} p_y r_{i.y}$$

where $DALY_{a_y,p_y,r_y}$ is the DALYs of age structure (a_y), population (p_y) and DALYs rate (r_y) in year y. $a_{i,y}$ is the proportion of population for the age group i in year y; p_y is the total population in year y; $r_{i,y}$ is DALYs rate in age group i in year y. The contribution of each factor to the changes of DALYs from 1990 to 2021 was defined by the effect of one factor changing while the others factors remain constant.

The effect of age structure, population, and DALYs rate from 1990 to 2021 can be obtained by the following three fomulas, respectively:

$$Effect_{a_{1990-2021}} = \left[\frac{DALY_{\boldsymbol{a_{2021}},p_{2021},r_{2021}} + DALY_{\boldsymbol{a_{2021}},p_{1990},r_{1990}}}{3} + \frac{DALY_{\boldsymbol{a_{2021}},p_{1990},r_{2021}} + DALY_{\boldsymbol{a_{2021}},p_{2021},r_{1990}}}{6}\right] - \left[\frac{DALY_{\boldsymbol{a_{1990}},p_{2021},r_{2021}} + DALY_{\boldsymbol{a_{1990}},p_{1990},r_{1990}}}{3} + \frac{DALY_{\boldsymbol{a_{1990}},p_{1990},r_{2021}} + DALY_{\boldsymbol{a_{1990}},p_{2021},r_{1990}}}{6}\right]$$

$$Effect_{p_{1990-2021}} = \left[\frac{DALY_{a_{2021},\boldsymbol{p_{2021}},r_{2021}} + DALY_{a_{1990},\boldsymbol{p_{2021}},r_{1990}}}{3} + \frac{DALY_{a_{1990},\boldsymbol{p_{2021}},r_{2021}} + DALY_{a_{2021},\boldsymbol{p_{2021}},r_{1990}}}{6}\right] - \left[\frac{DALY_{a_{2021},\boldsymbol{p_{1990}},r_{2021}} + DALY_{a_{1990},\boldsymbol{p_{1990}},r_{1990}}}{3} + \frac{DALY_{a_{1990},\boldsymbol{p_{1990}},r_{2021}} + DALY_{a_{2021},\boldsymbol{p_{1990}},r_{1990}}}{6}\right]$$

$$Effect_{r_{1990-202}} = \left[\frac{DALY_{a_{2021},p_{2021},\boldsymbol{r_{2021}}} + DALY_{a_{1990},p_{1990},\boldsymbol{r_{2021}}}}{3} + \frac{DALY_{a_{2021},p_{1990},\boldsymbol{r_{2021}}} + DALY_{a_{1990},p_{2021},\boldsymbol{r_{2021}}}}{6}\right] - \left[\frac{DALY_{a_{2021},p_{2021},\boldsymbol{r_{1990}}} + DALY_{a_{1990},p_{1990},\boldsymbol{r_{1990}}}}{3} + \frac{DALY_{a_{2021},p_{1990},\boldsymbol{r_{1990}}} + DALY_{a_{1990},p_{2021},\boldsymbol{r_{1990}}}}{6}\right]$$

Table S1. ICD codes used in CLD estimation

ICD 10 code (s)	ICD 9 code(s)
B18,B18.0,B18.1,B18.2,B18.8,B18.9,I85,I85.0,I85.00,I85.01,I85.1,I85.10,I85.11,I85.9,I98.2,K70,K70.0,K70.1,K70.10,K70.11,K70.2,K70.3,K70.30,K70.31,K71.7,K73,K73.0,K73.1,K73.2,K73.8,K73.9,K74,K74.0,K74.1,K74.2,K74.3,K74.4,K74.5,K74.6,K74.60,K74.69,K74.7,K74.8,K74.9,K75,K75.2,K75.4,K75.8,K75.81,K75.89,K75.9,K76,K76.0,K76.1,K76.2,K76.4,K76.5,K76.6,K76.7,K76.8,K76.81,K76.89,K76.9,K77.8	070.22,070.23,070.54,456.0,456.1,456.2,456.20,456.21,571,571.0,571.1,571.2,571.3,571.4,571.40,571.5,571.6,571.8,571.9,572.2,572.3,572.4,572.5,572.6,572.8,572.9,573,573.0,573.4,573.5,573.8,573.9

Table S2a. GBD locations of countries and territories in the East Asia Pacific region defined by the World Bank

Countries and territories	GBD location	SDI in 2021	SDI region
American Samoa	American Samoa	0.7237	High-middle
Australia	Australia	0.8443	High
Brunei Darussalam	Brunei Darussalam	0.8102	High-middle
Cambodia	Cambodia	0.4736	Low-middle
China	China	0.7216	High-middle
Hong Kong SAR, China			
Macao SAR, China			
Fiji	Fiji	0.6751	Middle
French Polynesia	*		
Guam	Guam	0.8040	High-middle
Indonesia	Indonesia	0.6569	Middle
Japan	Japan	0.8712	High
Kiribati	Kiribati	0.5272	Low-middle
Korea, Dem. People's Rep	North Korea	0.5699	Low-middle
Korea, Rep.	South Korea	0.8867	High
Laos PDR	Laos	0.4891	Low-middle
Malaysia	Malaysia	0.7425	High-middle
Marshall Islands	Marshall Islands	0.5741	Low-middle
Micronesia, Fed. Sts.	Micronesia (Federated States of)	0.5875	Low-middle
Mongolia	Mongolia	0.6176	Low-middle
Myanmar	Myanmar	0.5339	Low-middle
Nauru	Nauru	0.6252	Middle
New Caledonia	*		
New Zealand	New Zealand	0.8494	High
Northern Mariana Islands	Northern Mariana Islands	0.7715	High-middle
Palau	Palau	0.7540	High-middle
Papua New Guinea	Papua New Guinea	0.4178	Low
Philippines	Philippines	0.6512	Middle
Samoa	Samoa	0.5934	Low-middle

Singapore	Singapore	0.8561	High
Solomon Islands	Solomon Islands	0.4294	Low
Taiwan, China	Taiwan (Province of China)	0.8747	High
Thailand	Thailand	0.6825	Middle
Timor-Leste	Timor-Leste	0.4447	Low
Tonga	Tonga	0.6263	Middle
Tuvalu	Tuvalu	0.5766	Low-middle
Vanuatu	Vanuatu	0.4731	Low-middle
Vietnam	Viet Nam	0.6279	Middle

*: No corresponding in GBD locations

Table S2b. GBD 2021 quintile cutoffs

Quintile	Lower cutoff	Upper cutoff
Low SDI	0	0.4658
Low-middle SDI	0.4659	0.6188
Middle SDI	0.6189	0.7119
High-middle SDI	0.7102	0.8102
High SDI	0.8103	100

Table S2c. Corresponding rules for the calculation of the annual transition rate from CLD to liver cancer

Incident case	Prevalent case
Liver cancer due to hepatitis B	CLD due to hepatitis B
Liver cancer due to hepatitis C	CLD due to hepatitis C
Liver cancer due to alcohol use	CLD due to alcohol use
Liver cancer due to metabolic dysfunction–associated steatohepatitis	CLD due to MASLD
Liver cancer due to other causes	CLD due to other causes

Note: Although hepatoblastoma is included in the GBD liver cancer estimates, it was excluded from this analysis as it does not have a corresponding chronic liver disease etiology. Our transition analysis focused on etiologies that progress from chronic liver disease to liver cancer in adults, which does not apply to pediatric-specific cancers such as hepatoblastoma.

Table S2d. Proportion of HCC in non-cirrhotic patients

Etiology	Proportion of non-cirrhotic patients	Population	Reference
Hepatitis B	28.8% (45/156)	Taiwan	[22]
MASLD	65.2% (15/23)		
Hepatitis B	45.0% (90/200)	South Korea	[23]
MASLD	84.4% (27/32)		
MASLD	18.5% (10/54)	Singapore	[24]
Hepatitis B	9.4% (37/393)	South Korea	[25]
MASLD	25.0% (14/56)		
Hepatitis B	39.7% (345/869)	South Korea	[26]
Hepatitis C	34.3% (47/137)		
MASLD	52.3% (33/63)		
MASLD	73.3% (22/30)	Japan	[27]
MASLD	12.5% (1/8)	Japan	[28]
Hepatitis B	36.1% (22/61)	Japan	[29]
Hepatitis C	42.8% (63/147)		
MASLD	47.1% (8/17)		
Hepatitis B	37.8% (204/539)	South Korea	[30]
MASLD	47.2% (17/36)		
Hepatitis B	24.5%(297/1212)	97.6% from EAP including China, Taiwan, Korea, Japan, Vietnam, Cambodia, Thai	[31]
Hepatitis B	28.7%(259/900)	Taiwan	[32]
Hepatitis C	20.7%(162/781)		
MASLD*	31.0%(94/303)		
MASLD	27.6% (8/29)	Japan	[33]
Hepatitis B	19.2% (105/548)	South Korea	[34]
Hepatitis C	32.5% (13/40)		
MASLD*	48.7% (38/78)		
Hepatitis B	11.4% (73/638)	Philippines	[35]
Hepatitis C	13.3% (6/45)		
MASLD	12.2% (28/230)		
Hepatitis B	34.0% (73/215)	Thailand	[36]

*cryptogenic HCC is often considered a proxy for MASH-related HCC, particularly when viral and alcohol-related causes are excluded

Table S3. Prevalent cases and ASPR of CLD in 1990 and 2021, and temporal trend from 1990 to 2021.

Characteristics	1990		2021		1990-2021	
	**Prevalent cases No. × 10^6 (95% UI, %)**	**ASPR per 100,000 No. × 10^3(95% UI)**	**Prevalent cases No. × 10^6 (95% UI, %)**	**ASPR per 100,000 No. .× 10^3(95% UI)**	**Percentage change of prevalent cases No. (95% UI)**	**EAPC of ASPR No. (95% CI)**
Global	988.4 (919.5 to 1063.9,100.0%)	20.09 (18.74 to 21.51)	1697.3 (1575.3 to 1823.8,100.0%)	20.30 (18.85 to 21.79)	71.72% (67.41% to 75.53%)	0.01% (-0.03% to 0.05%)
**Sex**						
Male	529.3 (492.7 to 569.5,53.6%)	21.61 (20.18 to 23.19)	887.5 (825.2 to 950.9,52.3%)	21.54 (20.04 to 23.07)	67.66% (63.57% to 71.45%)	0.01% (-0.04% to 0.05%)
Female	459.1 (426.1 to 494.1,46.4%)	18.59 (17.32 to 19.92)	809.8 (750.3 to 871.5,47.7%)	19.07 (17.67 to 20.49)	76.40% (71.66% to 80.55%)	0.02% (-0.02% to 0.05%)
**SDI**						
High SDI	114.9 (106.8 to 123.4,11.6%)	11.59 (10.76 to 12.45)	197.3 (182.2 to 211.9,11.6%)	13.58 (12.54 to 14.62)	71.65% (68.25% to 74.87%)	0.58% (0.54% to 0.62%)
High-middle SDI	215.8 (200.8 to 231.2,21.8%)	20.10 (18.73 to 21.55)	329.6 (305.8 to 354.1,19.4%)	19.87 (18.41 to 21.37)	52.73% (47.94% to 56.70%)	-0.12% (-0.20% to -0.03%)

Middle SDI	354.5 (329.0 to 382.2,35.9%)	23.42 (21.87 to 25.16)	590.2 (546.6 to 634.6,34.8%)	21.78 (20.23 to 23.41)	66.50% (60.66% to 71.71%)	-0.28% (-0.35% to -0.20%)
Low-middle SDI	200.9 (187.1 to 216.2,20.3%)	21.52 (20.06 to 23.05)	376.7 (349.6 to 404.6,22.2%)	21.15 (19.65 to 22.72)	87.56% (82.94% to 91.85%)	-0.05% (-0.05% to -0.04%)
Low SDI	101.5 (94.6 to 109.6,10.3%)	25.21 (23.57 to 26.98)	202.1 (188.3 to 217.5,11.9%)	23.27 (21.69 to 24.95)	99.19% (94.75% to 103.44%)	-0.29% (-0.34% to -0.25%)
**Etiology**						
Hepatitis B	309.4 (282.4 to 340.5,31.3%)	5.73 (5.23 to 6.27)	283.6 (260.1 to 307.7,16.7%)	3.49 (3.20 to 3.79)	-8.32% (-11.70% to -4.64%)	-1.69% (-1.80% to -1.58%)
Hepatitis C	109.3 (89.3 to 131.7,11.1%)	2.17 (1.78 to 2.61)	138.6 (111.5 to 167.4,8.2%)	1.70 (1.37 to 2.06)	26.78% (21.87% to 31.50%)	-0.85% (-0.93% to -0.77%)
Alcohol use	1.7 (1.4 to 2.0,0.2%)	0.04 (0.03 to 0.05)	3.0 (2.5 to 3.5,0.2%)	0.03 (0.03 to 0.04)	73.69% (69.75% to 77.77%)	-0.53% (-0.61% to -0.45%)
MASLD	564.4 (516.5 to 618.1,57.1%)	12.08 (11.06 to 13.18)	1267.8 (1157.9 to 1380.4,74.7%)	15.02 (13.76 to 16.36)	124.62% (120.28% to 128.97%)	0.73% (0.67% to 0.79%)
Other causes	3.6 (3.0 to 4.1,0.4%)	0.06 (0.05 to 0.07)	4.2 (3.5 to 5.0,0.2%)	0.06 (0.05 to 0.06)	18.96% (15.55% to 23.07%)	-0.49% (-0.57% to -0.40%)
**Region**						

East Asia and Pacific	422.1 (393.0 to 454.6,42.7%)	23.79 (22.24 to 25.49)	607.0 (563.6 to 651.5,35.8%)	20.96 (19.50 to 22.50)	43.80% (38.23% to 48.77%)	-0.50% (-0.63% to -0.37%)
Europe and Central Asia	131.7 (122.1 to 141.5,13.3%)	13.99 (12.97 to 15.01)	189.2 (175.0 to 203.2,11.1%)	16.02 (14.81 to 17.30)	43.68% (41.08% to 46.09%)	0.44% (0.42% to 0.46%)
Latin America and Caribbean	63.6 (58.8 to 68.8,6.4%)	17.72 (16.40 to 19.09)	131.8 (121.8 to 142.5,7.8%)	18.54 (17.13 to 20.04)	107.17% (100.91% to 112.71%)	0.19% (0.17% to 0.20%)
Middle East and North Africa	57.2 (53.2 to 62.0,5.8%)	29.97 (27.88 to 32.17)	149.0 (138.2 to 161.2,8.8%)	32.39 (30.03 to 34.84)	160.30% (153.34% to 166.84%)	0.32% (0.27% to 0.37%)
North America	30.5 (28.1 to 33.1,3.1%)	9.61 (8.86 to 10.40)	55.2 (50.7 to 59.8,3.2%)	11.40 (10.51 to 12.32)	80.54% (76.07% to 84.96%)	0.65% (0.62% to 0.68%)
South Asia	164.4 (152.4 to 178.0,16.6%)	18.22 (16.91 to 19.58)	334.3 (309.3 to 360.8,19.7%)	18.30 (16.90 to 19.69)	103.33% (98.39% to 107.96%)	0.03% (-0.00% to 0.06%)
Sub-Saharan Africa	117.6 (109.6 to 126.8,11.9%)	28.40 (26.63 to 30.39)	229.0 (213.5 to 247.0,13.5%)	25.34 (23.70 to 27.10)	94.71% (90.30% to 99.25%)	-0.41% (-0.47% to -0.36%)

Table S4. DALYs and ASDR of CLD in 1990 and 2021, and temporal trend from 1990 to 2021.

Characteristics	1990		2021		1990-2021	
	DALYs No. × 10^3 (95% UI, %)	ASDR per 100,000 No. (95% UI)	DALYs No. × 10^3 (95% UI, %)	ASDR per 100,000 No. (95% UI)	Percentage change of DALYs No. (95% UI)	EAPC of ASDR No. (95% CI)
Global	36284.5 (33528.2 to 40328.3,100.0%)	799.94 (738.59 to 891.54)	46417.8 (43056.4 to 50687.9,100.0%)	545.07 (506.13 to 594.99)	27.93% (9.13% to 47.28%)	-1.27% (-1.37% to -1.16%)
**Sex**						
Male	24330.5 (22027.3 to 27526.5,67.1%)	1093.76 (990.39 to 1241.92)	31928.3 (29408.6 to 35408.4,68.8%)	762.35 (702.40 to 846.30)	31.23% (9.39% to 53.56%)	-1.19% (-1.30% to -1.09%)
Female	11954.0 (10531.1 to 13437.0,32.9%)	513.70 (452.58 to 578.72)	14489.5 (13105.5 to 16316.3,31.2%)	334.46 (302.86 to 375.82)	21.21% (2.10% to 46.24%)	-1.41% (-1.51% to -1.31%)
**SDI**						
High SDI	4665.5 (4505.0 to 4819.7,12.9%)	456.69 (440.91 to 471.85)	4985.5 (4800.1 to 5136.9,10.7%)	301.52 (292.07 to 310.26)	6.86% (2.78% to 11.72%)	-1.35% (-1.37% to -1.33%)
High-middle SDI	5728.8 (5350.8 to 6154.7,15.8%)	549.06 (512.61 to 589.87)	6581.0 (6133.0 to 7044.3,14.2%)	365.32 (340.63 to 390.84)	14.87% (3.53% to 27.68%)	-1.41% (-1.71% to -1.11%)

Middle SDI	11306.2 (10301.1 to 12876.3,31.2%)	860.02 (778.22 to 989.00)	14154.7 (13089.1 to 15420.3,30.5%)	511.82 (474.02 to 557.43)	25.19% (5.57% to 45.35%)	-1.79% (-1.83% to -1.75%)
Low-middle SDI	10308.1 (9076.3 to 12220.3,28.4%)	1273.22 (1116.33 to 1532.21)	14184.6 (12138.8 to 16260.4,30.6%)	846.05 (722.01 to 972.02)	37.61% (1.14% to 70.66%)	-1.22% (-1.35% to -1.08%)
Low SDI	4240.0 (3731.9 to 4741.7,11.7%)	1349.30 (1192.58 to 1530.89)	6470.3 (5703.1 to 7316.7,13.9%)	907.41 (807.08 to 1024.00)	52.60% (34.46% to 78.44%)	-1.32% (-1.45% to -1.19%)
**Etiology**						
Hepatitis B	12474.3 (10663.2 to 14629.5,34.4%)	279.53 (237.41 to 328.77)	13882.3 (11749.5 to 15998.4,29.9%)	161.92 (137.25 to 186.25)	11.29% (-8.53% to 32.39%)	-1.86% (-1.96% to -1.77%)
Hepatitis C	8630.2 (7148.3 to 10496.2,23.8%)	195.30 (162.76 to 236.70)	11822.9 (10109.2 to 13698.3,25.5%)	137.99 (117.99 to 159.91)	36.99% (16.14% to 57.13%)	-1.14% (-1.22% to -1.06%)
Alcohol use	7254.8 (6075.5 to 8534.1,20.0%)	167.64 (139.99 to 197.38)	11146.7 (9444.5 to 13296.8,24.0%)	128.39 (108.57 to 152.78)	53.65% (29.31% to 73.84%)	-0.89% (-1.03% to -0.75%)
MASLD	1285.2 (895.3 to 1775.5,3.5%)	30.57 (21.20 to 42.04)	2671.8 (1895.3 to 3602.0,5.8%)	30.90 (22.17 to 41.50)	107.88% (84.96% to 129.99%)	0.04% (-0.05% to 0.13%)
Other causes	6639.9 (5666.0 to 7746.7,18.3%)	126.90 (107.15 to 148.54)	6894.1 (5716.9 to 8275.7,14.9%)	85.86 (72.07 to 101.91)	3.83% (-9.47% to 22.16%)	-1.18% (-1.34% to -1.03%)
**Region**						

East Asia and Pacific	12327.9 (10908.3 to 14339.1,34.0%)	778.24 (685.67 to 908.39)	11805.0 (10419.0 to 13334.6,25.4%)	384.82 (340.62 to 431.91)	-4.24% (-23.14% to 16.21%)	-2.42% (-2.48% to -2.36%)
Europe and Central Asia	4811.1 (4713.3 to 4890.0,13.3%)	485.87 (476.35 to 494.16)	6162.9 (5878.5 to 6436.9,13.3%)	484.32 (460.72 to 506.53)	28.10% (22.05% to 33.82%)	-0.10% (-0.50% to 0.29%)
Latin America and Caribbean	2801.2 (2741.6 to 2857.8,7.7%)	862.23 (843.64 to 878.93)	4005.2 (3646.3 to 4328.0,8.6%)	556.39 (506.67 to 601.11)	42.98% (31.38% to 53.66%)	-1.53% (-1.58% to -1.47%)
Middle East and North Africa	1842.7 (1656.5 to 2058.2,5.1%)	1322.32 (1159.63 to 1494.83)	2336.0 (2020.3 to 2754.2,5.0%)	627.43 (544.73 to 736.59)	26.77% (4.11% to 53.91%)	-2.17% (-2.31% to -2.03%)
North America	1187.0 (1159.7 to 1206.5,3.3%)	375.38 (367.39 to 381.49)	2055.9 (1983.4 to 2117.7,4.4%)	386.97 (375.12 to 398.26)	73.20% (68.49% to 77.55%)	0.35% (0.26% to 0.43%)
South Asia	9078.0 (7889.1 to 10838.0,25.0%)	1079.71 (920.23 to 1321.64)	13089.4 (10421.5 to 15787.0,28.2%)	739.70 (585.54 to 892.43)	44.19% (-1.73% to 83.45%)	-1.17% (-1.32% to -1.03%)
Sub-Saharan Africa	4194.1 (3687.5 to 4824.7,11.6%)	1381.00 (1217.77 to 1628.89)	6913.9 (5778.3 to 8177.4,14.9%)	970.35 (831.41 to 1117.54)	64.85% (40.34% to 94.51%)	-1.15% (-1.25% to -1.06%)

Table S5. Death cases and age-standardized mortality rate (ASMR) of CLD in 1990 and 2021, and temporal trend from 1990 to 2021.

Characteristics	1990		2021		1990-2021	
	Death cases No. × 10^3 (95% UI, %)	ASMR per 100,000 No. (95% UI)	Death cases No. × 10^3 (95% UI, %)	ASMR per 100,000 No. (95% UI)	Percentage change of death cases No. (95% UI)	EAPC of ASMR No. (95% CI)
Global	1021.8 (936.6 to 1144.6,100.0%)	24.42 (22.38 to 27.48)	1425.1 (1308.1 to 1563.1,100.0%)	16.64 (15.28 to 18.26)	39.48% (16.85% to 61.31%)	-1.12% (-1.18% to -1.05%)
**Sex**						
Male	663.1 (599.7 to 754.8,64.9%)	33.19 (29.93 to 37.86)	947.4 (867.2 to 1046.8,66.5%)	23.13 (21.12 to 25.52)	42.87% (17.59% to 68.12%)	-1.06% (-1.12% to -0.99%)
Female	358.6 (312.6 to 408.3,35.1%)	16.40 (14.34 to 18.69)	477.7 (428.6 to 540.2,33.5%)	10.62 (9.55 to 12.00)	33.20% (10.15% to 64.30%)	-1.27% (-1.33% to -1.21%)
**SDI**						
High SDI	151.4 (146.0 to 156.6,14.8%)	14.31 (13.79 to 14.80)	181.9 (171.0 to 188.7,12.8%)	9.79 (9.34 to 10.11)	20.14% (14.19% to 25.46%)	-1.27% (-1.30% to -1.24%)
High-middle SDI	177.8 (166.0 to 190.2,17.4%)	17.65 (16.49 to 18.88)	215.0 (200.0 to 230.8,15.1%)	11.38 (10.60 to 12.20)	20.94% (8.46% to 33.85%)	-1.22% (-1.39% to -1.06%)
Middle SDI	305.4 (275.0 to 353.9,29.9%)	27.14 (24.17 to 31.76)	441.5 (403.4 to 486.0,31.0%)	16.43 (14.98 to 18.09)	44.56% (19.26% to 70.24%)	-1.61% (-1.65% to -1.57%)

Low-middle SDI	275.2 (239.9 to 333.0,26.9%)	41.70 (36.19 to 50.94)	414.1 (351.5 to 476.3,29.1%)	27.45 (23.17 to 31.55)	50.49% (7.82% to 89.79%)	-1.09% (-1.17% to -1.01%)
Low SDI	111.0 (98.2 to 125.7,10.9%)	43.62 (38.23 to 50.39)	171.3 (152.7 to 193.0,12.0%)	29.74 (26.54 to 33.48)	54.39% (33.74% to 81.53%)	-0.98% (-1.08% to -0.89%)
**Etiology**						
Hepatitis B	359.8 (306.1 to 423.8,35.2%)	8.60 (7.29 to 10.11)	432.0 (365.2 to 502.4,30.3%)	5.03 (4.26 to 5.85)	20.04% (-2.42% to 43.05%)	-1.65% (-1.73% to -1.58%)
Hepatitis C	257.0 (214.9 to 316.0,25.2%)	6.26 (5.23 to 7.65)	368.3 (314.0 to 427.5,25.8%)	4.29 (3.67 to 4.98)	43.29% (19.82% to 65.59%)	-1.14% (-1.19% to -1.09%)
Alcohol use	223.0 (185.5 to 263.0,21.8%)	5.40 (4.52 to 6.39)	354.3 (299.2 to 418.9,24.9%)	4.08 (3.45 to 4.81)	58.85% (33.50% to 78.72%)	-0.69% (-0.78% to -0.60%)
MASLD	44.9 (31.7 to 61.0,4.4%)	1.16 (0.81 to 1.57)	97.4 (69.5 to 130.2,6.8%)	1.14 (0.82 to 1.52)	117.12% (91.64% to 141.72%)	0.08% (0.03% to 0.13%)
Other causes	137.1 (112.3 to 164.3,13.4%)	3.00 (2.42 to 3.69)	173.2 (137.4 to 214.5,12.2%)	2.09 (1.67 to 2.56)	26.41% (6.02% to 47.92%)	-0.93% (-1.01% to -0.85%)
**Region**						
East Asia and Pacific	348.5 (305.5 to 409.7,34.1%)	24.71 (21.59 to 29.23)	390.2 (343.7 to 440.9,27.4%)	12.28 (10.83 to 13.83)	11.95% (-11.30% to 37.46%)	-2.23% (-2.29% to -2.18%)

Europe and Central Asia	157.8 (153.6 to 160.6,15.4%)	15.31 (14.91 to 15.60)	200.0 (189.9 to 208.6,14.0%)	14.19 (13.51 to 14.82)	26.77% (20.86% to 32.07%)	-0.02% (-0.23% to 0.19%)
Latin America and Caribbean	77.1 (75.3 to 78.6,7.5%)	26.35 (25.61 to 26.89)	128.1 (116.2 to 138.5,9.0%)	17.96 (16.28 to 19.43)	66.11% (52.73% to 78.12%)	-1.33% (-1.36% to -1.30%)
Middle East and North Africa	64.1 (56.0 to 73.0,6.3%)	59.79 (50.92 to 68.91)	84.3 (73.3 to 98.8,5.9%)	26.19 (22.80 to 30.38)	31.50% (7.55% to 62.09%)	-2.40% (-2.50% to -2.31%)
North America	37.6 (36.3 to 38.4,3.7%)	11.43 (11.06 to 11.63)	71.6 (67.8 to 73.9,5.0%)	12.24 (11.68 to 12.62)	90.17% (84.63% to 95.00%)	-0.08% (-0.25% to 0.09%)
South Asia	222.0 (188.4 to 273.8,21.7%)	31.68 (26.25 to 40.03)	363.9 (281.1 to 443.1,25.5%)	22.75 (17.43 to 27.70)	63.92% (4.87% to 115.95%)	-0.76% (-0.86% to -0.66%)
Sub-Saharan Africa	113.3 (99.8 to 133.7,11.1%)	46.07 (40.06 to 55.33)	185.5 (158.9 to 213.5,13.0%)	32.67 (28.73 to 36.82)	63.63% (39.77% to 90.06%)	-0.91% (-0.99% to -0.83%)

Table S6. Prevalent cases and ASPR of CLD in EAP in 1990 and 2021, and temporal trend from 1990 to 2021.

Characteristics	1990		2021		1990-2021	
	Prevalent cases No. $\times 10^3$ (95% UI, %)	ASPR per 100,000 No. $\times 10^3$(95% UI)	Prevalent cases No. $\times 10^3$ (95% UI, %)	ASPR per 100,000 No. .$\times 10^3$(95% UI)	Percentage change of prevalent cases No. (95% UI)	EAPC of ASPR No. (95% CI)
East Asia and Pacific	422115.8 (393014.8 to 454629.9,100.0%)	23.79 (22.24 to 25.49)	606998.6 (563588.3 to 651478.0,100.0%)	20.96 (19.50 to 22.50)	43.80% (38.23% to 48.77%)	-0.50% (-0.63% to -0.37%)
**Sex**						
Male	227067.6 (211722.2 to 244652.5,53.8%)	25.24 (23.62 to 26.99)	309755.8 (288868.7 to 332188.7,51.0%)	21.64 (20.17 to 23.20)	36.42% (31.47% to 40.92%)	-0.49% (-0.63% to -0.34%)
Female	195048.2 (181307.1 to 210151.0,46.2%)	22.30 (20.76 to 23.91)	297242.8 (274632.7 to 319591.9,49.0%)	20.25 (18.74 to 21.80)	52.39% (45.76% to 58.08%)	-0.52% (-0.64% to -0.40%)
**SDI***						
High SDI	31920.7 (29746.7 to 34110.6,7.6%)	13.65 (12.76 to 14.57)	44474.6 (41191.3 to 47785.5,7.3%)	12.77 (11.85 to 13.74)	39.33%	-0.22% (-0.28% to -0.15%)
High-middle SDI	295200.3 (274875.1 to 319128.9,69.9%)	25.80 (24.11 to 27.70)	402394.8 (373401.7 to 432290.5,66.3%)	21.70 (20.20 to 23.33)	36.31%	-0.69% (-0.87% to -0.51%)

Middle SDI	77441.0 (71947.5 to 83371.7,18.3%)	24.34 (22.68 to 26.06)	132294.4 (122704.6 to 142371.5,21.8%)	22.36 (20.80 to 24.00)	70.83%	-0.29% (-0.30% to -0.28%)
Low-middle SDI	16285.3 (14926.5 to 17756.6,3.9%)	24.00 (22.11 to 26.00)	25143.3 (23180.7 to 27200.6,4.1%)	22.11 (20.39 to 23.87)	54.39%	-0.30% (-0.33% to -0.27%)
Low SDI	1268.6 (1181.4 to 1371.0,0.3%)	28.89 (27.08 to 30.84)	2691.5 (2517.0 to 2904.2,0.4%)	25.84 (24.23 to 27.63)	112.16%	-0.39% (-0.40% to -0.37%)
**Etiology**						
Hepatitis B	169924.1 (155089.2 to 186869.8,40.3%)	9.00 (8.24 to 9.84)	121235.1 (110299.6 to 132139.3,20.0%)	4.38 (3.97 to 4.78)	-28.65% (-32.73% to -24.46%)	-2.53% (-2.64% to -2.43%)
Hepatitis C	44475.6 (36478.6 to 53908.7,10.5%)	2.47 (2.03 to 2.98)	42400.1 (34297.3 to 51419.5,7.0%)	1.65 (1.33 to 2.01)	-4.67% (-9.06% to -0.02%)	-1.63% (-1.92% to -1.33%)
Alcohol use	356.2 (288.1 to 429.9,0.1%)	0.02 (0.02 to 0.03)	571.2 (459.0 to 690.1,0.1%)	0.02 (0.01 to 0.02)	60.36% (53.45% to 67.71%)	-1.02% (-1.06% to -0.97%)
MASLD	206658.6 (189009.3 to 227071.1,49.0%)	12.27 (11.21 to 13.42)	442307.1 (403792.6 to 481689.0,72.9%)	14.89 (13.61 to 16.23)	114.03% (106.52% to 121.93%)	0.66% (0.51% to 0.81%)
Other causes	701.3 (571.1 to 848.9,0.2%)	0.04 (0.03 to 0.04)	485.1 (392.2 to 578.4,0.1%)	0.02 (0.02 to 0.03)	-30.82% (-35.60% to -24.56%)	-1.57% (-1.66% to -1.48%)
**Country or territory**						

China	291845.4 (271775.8 to 315478.8,69.1%)	25.84 (24.16 to 27.75)	394433.3 (366030.7 to 423675.1,65.0%)	21.66 (20.16 to 23.28)	35.15% (29.09% to 40.59%)	-0.71% (-0.89% to -0.52%)
Indonesia	36596.0 (33908.7 to 39567.2,8.7%)	23.69 (21.97 to 25.45)	67900.3 (62832.4 to 73241.1,11.2%)	23.36 (21.68 to 25.14)	85.54% (79.78% to 91.06%)	-0.05% (-0.06% to -0.04%)
Japan	16955.0 (15723.5 to 18140.8,4.0%)	11.26 (10.47 to 12.05)	21002.1 (19464.6 to 22571.2,3.5%)	10.69 (9.89 to 11.52)	23.87% (19.08% to 28.39%)	-0.34% (-0.49% to -0.20%)
Philippines	13507.5 (12413.4 to 14635.0,3.2%)	25.76 (23.93 to 27.69)	22845.2 (21181.1 to 24572.8,3.8%)	21.86 (20.34 to 23.40)	69.13% (62.32% to 76.38%)	-0.56% (-0.57% to -0.54%)
Viet Nam	15770.7 (14741.8 to 16834.1,3.7%)	26.82 (25.20 to 28.47)	23428.8 (21888.8 to 25153.0,3.9%)	21.72 (20.38 to 23.23)	48.56% (40.85% to 55.77%)	-0.70% (-0.71% to -0.69%)
Thailand	11415.3 (10744.2 to 12169.9,2.7%)	21.95 (20.60 to 23.40)	17889.5 (16590.1 to 19154.4,2.9%)	19.96 (18.53 to 21.37)	56.71% (49.97% to 63.87%)	-0.34% (-0.39% to -0.29%)
Myanmar	6819.3 (6277.1 to 7411.3,1.6%)	20.50 (18.89 to 22.20)	11443.6 (10549.6 to 12427.5,1.9%)	20.62 (19.00 to 22.33)	67.81% (61.99% to 73.83%)	0.02% (0.01% to 0.03%)
South Korea	6186.3 (5754.8 to 6644.5,1.5%)	14.37 (13.45 to 15.38)	9579.0 (8855.3 to 10303.9,1.6%)	12.96 (12.07 to 13.90)	54.84% (46.79% to 63.75%)	-0.08% (-0.25% to 0.10%)
Malaysia	3276.4 (3026.8 to 3564.7,0.8%)	22.94 (21.25 to 24.72)	7817.9 (7238.6 to 8460.4,1.3%)	23.79 (22.03 to 25.71)	138.61% (130.20% to 146.19%)	0.12% (0.10% to 0.14%)

North Korea	5501.7 (5030.6 to 5991.2,1.3%)	27.47 (25.21 to 29.76)	7643.3 (7064.7 to 8205.9,1.3%)	24.55 (22.67 to 26.36)	38.93% (31.24% to 46.93%)	-0.51% (-0.58% to -0.44%)
Taiwan (Province of China)	5712.2 (5425.4 to 6014.9,1.4%)	28.37 (26.92 to 29.87)	7893.1 (7344.6 to 8460.4,1.3%)	23.67 (22.14 to 25.39)	38.18% (33.02% to 43.48%)	-0.50% (-0.54% to -0.46%)
Australia	2155.1 (1995.1 to 2328.0,0.5%)	11.80 (10.97 to 12.74)	3985.6 (3667.8 to 4276.4,0.7%)	12.12 (11.14 to 13.09)	84.94% (78.68% to 91.86%)	0.09% (0.04% to 0.13%)
Cambodia	2509.0 (2289.2 to 2756.7,0.6%)	29.61 (27.44 to 32.08)	3718.2 (3428.8 to 4021.8,0.6%)	23.22 (21.44 to 24.96)	48.20% (39.56% to 57.04%)	-0.78% (-0.82% to -0.74%)
Papua New Guinea	1057.2 (989.8 to 1137.1,0.3%)	30.13 (28.40 to 32.08)	2323.9 (2177.6 to 2506.0,0.4%)	26.58 (24.99 to 28.38)	119.81% (110.51% to 129.84%)	-0.44% (-0.46% to -0.42%)
Laos	788.7 (723.6 to 863.2,0.2%)	22.85 (21.17 to 24.70)	1326.4 (1226.4 to 1427.7,0.2%)	19.86 (18.43 to 21.39)	68.17% (59.58% to 77.40%)	-0.51% (-0.53% to -0.49%)
Singapore	496.4 (462.2 to 534.9,0.1%)	15.85 (14.77 to 16.94)	1221.6 (1125.1 to 1319.0,0.2%)	15.82 (14.64 to 17.08)	146.10% (134.67% to 157.23%)	-0.02% (-0.04% to -0.00%)
New Zealand	415.7 (385.7 to 447.4,0.1%)	11.39 (10.56 to 12.31)	793.2 (733.9 to 854.7,0.1%)	12.33 (11.40 to 13.31)	90.81% (84.39% to 97.36%)	0.25% (0.25% to 0.26%)
Mongolia	564.3 (512.1 to 622.9,0.1%)	31.94 (29.26 to 34.72)	843.1 (755.0 to 935.1,0.1%)	26.73 (24.12 to 29.47)	49.39% (40.61% to 58.53%)	-0.28% (-0.42% to -0.15%)

Timor-Leste	143.8 (131.1 to 159.2,0.0%)	23.45 (21.50 to 25.40)	243.3 (224.7 to 263.5,0.0%)	21.48 (19.82 to 23.27)	69.20% (60.52% to 77.69%)	-0.29% (-0.31% to -0.28%)
Fiji	130.3 (119.9 to 142.4,0.0%)	21.77 (20.02 to 23.59)	207.7 (190.8 to 225.6,0.0%)	23.24 (21.38 to 25.23)	59.42% (52.68% to 65.62%)	0.20% (0.16% to 0.23%)
Solomon Islands	67.6 (60.5 to 74.8,0.0%)	26.41 (24.06 to 28.52)	124.3 (114.7 to 134.8,0.0%)	23.36 (21.65 to 25.05)	83.76% (69.77% to 96.62%)	-0.34% (-0.40% to -0.29%)
Brunei Darussalam	31.7 (29.4 to 34.4,0.0%)	14.95 (13.90 to 16.08)	73.4 (67.7 to 79.5,0.0%)	15.34 (14.12 to 16.48)	131.11% (119.26% to 141.11%)	0.16% (0.13% to 0.20%)
Vanuatu	29.6 (27.1 to 32.3,0.0%)	26.20 (24.19 to 28.35)	65.5 (60.8 to 70.8,0.0%)	26.00 (24.16 to 27.83)	121.19% (108.82% to 133.05%)	-0.01% (-0.06% to 0.04%)
Samoa	29.5 (27.2 to 32.0,0.0%)	23.36 (21.60 to 25.21)	40.7 (37.7 to 43.9,0.0%)	23.03 (21.34 to 24.84)	38.09% (32.82% to 44.14%)	-0.08% (-0.12% to -0.05%)
Guam	25.0 (23.2 to 27.3,0.0%)	21.14 (19.67 to 22.83)	39.3 (36.3 to 42.2,0.0%)	21.41 (19.87 to 22.98)	57.15% (48.69% to 64.67%)	0.05% (0.02% to 0.08%)
Kiribati	16.6 (15.2 to 18.2,0.0%)	27.62 (25.50 to 30.01)	28.1 (26.0 to 30.6,0.0%)	27.15 (25.16 to 29.34)	69.06% (61.54% to 77.16%)	-0.12% (-0.17% to -0.07%)
Micronesia (Federated States of)	17.4 (16.0 to 19.0,0.0%)	22.77 (21.04 to 24.59)	20.8 (19.2 to 22.6,0.0%)	22.33 (20.69 to 24.19)	19.54% (14.23% to 25.53%)	-0.09% (-0.13% to -0.06%)

Tonga	19.6 (17.9 to 21.2,0.0%)	25.74 (23.79 to 27.69)	21.2 (19.7 to 22.7,0.0%)	23.82 (22.18 to 25.53)	8.15% (3.18% to 14.12%)	-0.34% (-0.39% to -0.29%)
Northern Mariana Islands	9.9 (9.1 to 10.8,0.0%)	24.82 (23.12 to 26.76)	13.3 (12.3 to 14.4,0.0%)	24.30 (22.48 to 26.21)	34.65% (27.25% to 42.77%)	-0.11% (-0.16% to -0.07%)
American Samoa	8.8 (8.1 to 9.6,0.0%)	23.88 (22.25 to 25.68)	12.5 (11.6 to 13.4,0.0%)	24.91 (23.14 to 26.66)	41.75% (34.23% to 49.02%)	0.12% (0.06% to 0.17%)
Marshall Islands	7.1 (6.5 to 7.8,0.0%)	22.65 (20.95 to 24.53)	10.9 (10.0 to 11.8,0.0%)	21.90 (20.16 to 23.68)	52.85% (43.67% to 61.20%)	-0.12% (-0.17% to -0.07%)
Palau	3.0 (2.7 to 3.2,0.0%)	21.92 (20.31 to 23.66)	5.1 (4.6 to 5.5,0.0%)	22.21 (20.55 to 23.90)	70.78% (61.82% to 79.29%)	0.04% (-0.00% to 0.08%)
Nauru	1.7 (1.5 to 1.9,0.0%)	22.01 (20.21 to 23.91)	1.8 (1.6 to 1.9,0.0%)	20.71 (19.10 to 22.30)	4.07% (-0.86% to 9.21%)	-0.20% (-0.22% to -0.17%)
Tuvalu	1.9 (1.8 to 2.1,0.0%)	23.22 (21.32 to 25.05)	2.6 (2.4 to 2.8,0.0%)	22.77 (21.18 to 24.49)	36.01% (29.04% to 43.08%)	-0.03% (-0.08% to 0.01%)

Countries and territories sorted by population in 2021. *: Since there is no predefined SDI region for the East Asia and Pacific (EAP) region, we calculated the Age-Standardized Rate (ASR) for each SDI region by using the population and respective values for each Country or territory within the specific SDI region. The cases represent the cumulative sum of the respective values for each Country or territory within the specific SDI region.

Table S7. DALYs and ASDR of CLD in EAP in 1990 and 2021, and temporal trend from 1990 to 2021.

Characteristics	1990		2021		1990-2021	
	DALYs No. $\times 10^3$ (95% UI,%	ASDR per 100,000 No. (95% UI)	DALYs No. $\times 10^3$ (95% UI,%)	ASDR per 100,000 No. (95% UI)	Percentage change of DALYs No. (95% UI)	EAPC of ASDR No. (95% CI)
East Asia and Pacific	12327.9 (10908.3 to 14339.1,100.0 %)	778.24 (685.67 to 908.39)	11805.0 (10419.0 to 13334.6,100.0%)	384.82 (340.62 to 431.91)	-4.24% (-23.14% to 16.21%)	-2.42% (-2.48% to -2.36%)
**Sex**						
Male	8420.6 (7325.2 to 9915.4,68.3%)	1055.23 (916.79 to 1249.35)	8600.3 (7407.3 to 10014.8,72.9%)	565.53 (488.42 to 657.61)	2.13% (-20.48% to 30.59%)	-2.14% (-2.20% to -2.09%)
Female	3907.3 (3235.2 to 4610.6,31.7%)	498.49 (411.42 to 588.91)	3204.8 (2755.5 to 3821.7,27.1%)	205.38 (177.36 to 243.41)	-17.98% (-34.83% to 9.74%)	-3.03% (-3.11% to -2.96%)
**SDI***						
High SDI	1446.6 (1294.8 to 1520.1,11.7%)	658.71 (584.15 to 694.75)	942.6 (859.5 to 1038.4,8.0%)	224.03 (208.25 to 245.66)	-34.84%	-3.62% (-3.71% to -3.53%)
High-middle SDI	6297.4 (5340.0 to 7306.8,51.1%)	629.36 (534.52 to 729.55)	4651.2 (3680.2 to 5724.8,39.4%)	229.31 (182.27 to 281.16)	-26.14%	-3.51% (-3.62% to -3.40%)
Middle SDI	3686.7 (2920.2 to 4886.7,29.9%)	1368.15 (1060.13 to 1857.06)	5188.2 (4187.5 to 6457.4,43.9%)	916.09 (742.85 to 1120.71)	40.73%	-1.29% (-1.35% to -1.22%)

Low-middle SDI	880.3 (600.7 to 1270.1,7.1%)	1513.96 (1047.22 to 2225.09)	990.8 (690.7 to 1324.0,8.4%)	903.45 (638.52 to 1198.25)	12.55%	-1.90% (-1.99% to -1.81%)
Low SDI	16.9 (10.7 to 26.6,0.1%)	490.38 (310.08 to 792.71)	32.2 (24.2 to 44.1,0.3%)	344.22 (257.07 to 472.28)	90.71%	-1.24% (-1.39% to -1.08%)
**Etiology**						
Hepatitis B	6705.1 (5750.5 to 7793.8,54.4%)	424.75 (365.00 to 493.33)	5640.8 (4672.4 to 6666.0,47.8%)	180.05 (150.31 to 212.05)	-15.87% (-34.45% to 5.07%)	-3.01% (-3.11% to -2.90%)
Hepatitis C	2570.9 (2075.4 to 3304.9,20.9%)	164.74 (133.52 to 211.48)	2840.6 (2388.9 to 3425.2,24.1%)	92.30 (77.58 to 111.82)	10.49% (-13.57% to 35.24%)	-1.92% (-1.98% to -1.87%)
Alcohol use	1442.2 (1138.4 to 1799.0,11.7%)	95.46 (75.48 to 119.59)	1929.2 (1576.7 to 2442.8,16.3%)	59.13 (48.48 to 74.54)	33.77% (5.21% to 60.52%)	-1.59% (-1.65% to -1.53%)
MASLD	255.5 (170.7 to 363.2,2.1%)	17.63 (11.91 to 24.78)	411.5 (285.4 to 571.6,3.5%)	12.85 (9.07 to 17.56)	61.05% (31.59% to 100.15%)	-1.03% (-1.14% to -0.92%)
Other causes	1354.2 (1108.6 to 1660.7,11.0%)	75.66 (62.45 to 93.55)	983.0 (776.9 to 1227.4,8.3%)	40.50 (32.93 to 49.18)	-27.41% (-40.47% to -9.94%)	-2.03% (-2.07% to -2.00%)
**Country and territory**						
China	6236.6 (5286.2 to 7236.7,50.6%)	631.12 (535.68 to 731.75)	4500.1 (3552.3 to 5555.1,38.1%)	223.63 (177.20 to 275.33)	-27.84% (-46.46% to -3.75%)	-3.60% (-3.71% to -3.48%)

Indonesia	2532.4 (2017.8 to 3334.1,20.5%)	1881.13 (1462.95 to 2537.19)	3276.2 (2692.8 to 4051.0,27.8%)	1219.68 (1004.23 to 1479.01)	29.37% (-7.65% to 74.24%)	-1.35% (-1.44% to -1.26%)
Japan	649.6 (631.3 to 663.5,5.3%)	386.97 (375.74 to 395.37)	463.3 (423.2 to 490.9,3.9%)	184.52 (174.95 to 192.16)	-28.68% (-33.08% to - 24.85%)	-2.51% (-2.63% to -2.38%)
Philippines	299.4 (247.3 to 393.3,2.4%)	684.79 (547.56 to 943.58)	507.4 (421.0 to 613.9,4.3%)	512.79 (425.72 to 616.41)	69.45% (16.31% to 125.33%)	-0.92% (-0.97% to -0.88%)
Viet Nam	490.1 (359.5 to 706.8,4.0%)	1095.29 (796.11 to 1601.03)	738.1 (551.8 to 941.8,6.3%)	682.34 (511.62 to 865.21)	50.59% (-13.26% to 131.74%)	-1.51% (-1.58% to -1.43%)
Thailand	361.7 (293.2 to 448.7,2.9%)	796.06 (643.51 to 981.44)	663.3 (519.5 to 846.5,5.6%)	691.80 (543.68 to 873.41)	83.36% (29.68% to 152.52%)	-0.49% (-0.67% to -0.31%)
Myanmar	508.2 (321.6 to 755.0,4.1%)	1629.04 (1037.62 to 2459.71)	516.4 (360.4 to 674.0,4.4%)	909.98 (642.81 to 1182.18)	1.62% (-34.42% to 48.24%)	-2.19% (-2.31% to -2.07%)
South Korea	574.9 (449.9 to 626.0,4.7%)	1533.00 (1225.76 to 1661.84)	247.7 (223.1 to 299.4,2.1%)	288.47 (260.63 to 350.24)	-56.92% (-62.76% to - 35.56%)	-5.71% (-5.91% to -5.52%)
Malaysia	58.9 (52.2 to 67.8,0.5%)	513.90 (459.28 to 584.35)	148.4 (125.6 to 166.5,1.3%)	480.33 (406.13 to 539.13)	151.91% (98.97% to 199.71%)	-0.74% (-0.97% to -0.51%)
North Korea	117.3 (87.1 to 163.3,1.0%)	620.09 (466.84 to 862.74)	153.2 (96.1 to 220.4,1.3%)	453.40 (289.62 to 649.82)	30.57% (-17.33% to 100.43%)	-1.03% (-1.08% to -0.97%)

Taiwan (Province of China)	171.3 (165.1 to 177.5,1.4%)	954.79 (920.43 to 990.03)	165.2 (151.4 to 177.5,1.4%)	445.82 (410.89 to 476.81)	-3.58% (-11.56% to 5.09%)	-2.62% (-2.84% to -2.40%)
Australia	39.5 (37.8 to 41.5,0.3%)	211.93 (202.46 to 221.90)	55.9 (52.0 to 59.5,0.5%)	153.83 (144.19 to 163.34)	41.48% (30.60% to 52.11%)	-0.64% (-0.82% to -0.45%)
Cambodia	185.0 (138.7 to 260.0,1.5%)	2844.60 (2168.58 to 4129.43)	241.8 (174.3 to 320.6,2.0%)	1624.07 (1167.81 to 2138.90)	30.70% (-19.09% to 91.73%)	-2.05% (-2.13% to -1.96%)
Papua New Guinea	10.7 (6.8 to 16.1,0.1%)	380.11 (243.91 to 572.12)	21.1 (16.4 to 27.3,0.2%)	266.85 (205.90 to 343.91)	96.59% (23.74% to 208.77%)	-1.26% (-1.42% to -1.10%)
Laos	42.4 (30.5 to 58.4,0.3%)	1546.56 (1112.30 to 2242.17)	46.7 (33.3 to 69.2,0.4%)	784.31 (561.48 to 1148.47)	10.12% (-22.68% to 56.91%)	-2.43% (-2.54% to -2.31%)
Singapore	6.9 (6.6 to 7.2,0.1%)	259.48 (248.44 to 270.85)	4.5 (4.2 to 4.8,0.0%)	53.56 (49.68 to 56.91)	-34.49% (-39.54% to -29.47%)	-5.01% (-5.12% to -4.90%)
New Zealand	4.3 (4.1 to 4.5,0.0%)	116.80 (110.90 to 122.46)	6.0 (5.6 to 6.3,0.1%)	82.24 (77.23 to 86.77)	38.54% (28.82% to 49.40%)	-0.94% (-1.13% to -0.74%)
Mongolia	23.9 (20.4 to 28.3,0.2%)	1720.77 (1476.16 to 2054.07)	27.4 (23.1 to 32.3,0.2%)	968.48 (819.45 to 1134.07)	15.00% (-8.42% to 43.99%)	-1.64% (-2.03% to -1.25%)
Timor-Leste	4.1 (2.7 to 7.4,0.0%)	845.88 (521.19 to 1599.00)	6.1 (4.1 to 10.2,0.1%)	622.58 (414.35 to 1047.92)	48.45% (4.08% to 115.14%)	-1.11% (-1.36% to -0.87%)

Fiji	2.3 (1.8 to 2.9,0.0%)	401.39 (309.62 to 522.35)	2.5 (1.9 to 3.3,0.0%)	277.41 (205.87 to 360.85)	11.11% (-27.70% to 62.12%)	-1.28% (-1.41% to -1.16%)
Solomon Islands	2.0 (1.2 to 3.1,0.0%)	996.70 (619.02 to 1586.84)	5.0 (3.7 to 6.5,0.0%)	958.80 (718.16 to 1259.25)	145.65% (51.61% to 285.10%)	-0.04% (-0.11% to 0.04%)
Brunei Darussalam	0.5 (0.4 to 0.6,0.0%)	356.30 (294.87 to 418.00)	1.0 (0.9 to 1.2,0.0%)	223.84 (191.56 to 260.18)	96.00% (56.62% to 143.08%)	-1.32% (-1.56% to -1.08%)
Vanuatu	1.1 (0.6 to 2.0,0.0%)	1173.95 (646.83 to 2098.22)	2.2 (1.5 to 3.4,0.0%)	904.05 (614.06 to 1406.12)	96.29% (34.94% to 189.23%)	-0.99% (-1.08% to -0.90%)
Samoa	0.7 (0.5 to 1.0,0.0%)	601.26 (420.24 to 887.19)	0.9 (0.6 to 1.2,0.0%)	497.46 (346.71 to 666.59)	27.47% (-9.13% to 76.08%)	-0.65% (-0.85% to -0.45%)
Guam	0.8 (0.7 to 1.0,0.0%)	741.24 (647.81 to 888.03)	1.0 (0.9 to 1.2,0.0%)	551.31 (477.92 to 625.09)	29.92% (-1.09% to 58.95%)	-0.64% (-0.91% to -0.37%)
Kiribati	0.6 (0.5 to 0.8,0.0%)	1115.56 (838.95 to 1493.80)	1.0 (0.7 to 1.3,0.0%)	918.82 (667.75 to 1229.71)	56.18% (7.67% to 116.31%)	-0.62% (-0.69% to -0.55%)
Micronesia (Federated States of)	0.7 (0.5 to 0.9,0.0%)	1066.34 (802.64 to 1382.07)	0.7 (0.5 to 1.0,0.0%)	714.60 (478.87 to 991.81)	-4.76% (-40.29% to 43.30%)	-1.33% (-1.44% to -1.22%)
Tonga	0.7 (0.5 to 0.9,0.0%)	1004.54 (790.92 to 1293.26)	0.7 (0.5 to 0.9,0.0%)	737.80 (528.48 to 1005.51)	-3.46% (-41.14% to 44.49%)	-0.93% (-0.96% to -0.89%)

Northern Mariana Islands	0.3 (0.2 to 0.4,0.0%)	970.82 (757.03 to 1247.58)	0.4 (0.3 to 0.4,0.0%)	672.06 (582.03 to 754.33)	17.72% (-15.40% to 63.11%)	-1.26% (-1.54% to -0.98%)
American Samoa	0.2 (0.1 to 0.2,0.0%)	507.12 (405.42 to 621.52)	0.2 (0.2 to 0.3,0.0%)	413.05 (335.85 to 510.02)	26.77% (-8.07% to 75.34%)	-0.73% (-0.96% to -0.50%)
Marshall Islands	0.3 (0.2 to 0.4,0.0%)	1183.95 (881.49 to 1528.66)	0.4 (0.3 to 0.6,0.0%)	842.72 (528.87 to 1198.02)	39.79% (-24.94% to 111.61%)	-1.14% (-1.18% to -1.10%)
Palau	0.1 (0.1 to 0.1,0.0%)	707.71 (471.78 to 967.03)	0.1 (0.1 to 0.2,0.0%)	559.93 (379.92 to 737.86)	45.34% (7.38% to 99.36%)	-0.68% (-0.74% to -0.61%)
Nauru	0.1 (0.1 to 0.1,0.0%)	1151.32 (710.12 to 1487.02)	0.1 (0.0 to 0.1,0.0%)	836.88 (412.03 to 1199.72)	-13.02% (-43.64% to 21.15%)	-1.11% (-1.30% to -0.92%)
Tuvalu	0.1 (0.1 to 0.1,0.0%)	1030.45 (775.41 to 1311.71)	0.1 (0.1 to 0.1,0.0%)	667.12 (518.88 to 839.95)	-7.28% (-32.09% to 25.39%)	-1.34% (-1.45% to -1.24%)

Countries and territories sorted by population in 2021. *: Since there is no predefined SDI region for the East Asia and Pacific (EAP) region, we calculated the Age-Standardized Rate (ASR) for each SDI region by using the population and respective values for each Country or territory within the specific SDI region. The cases represent the cumulative sum of the respective values for each Country or territory within the specific SDI region.

Table S8. Death cases and ASMR of CLD in EAP in 1990 and 2021, and temporal trend from 1990 to 2021.

Characteristics	1990		2021		1990-2021	
	Death cases No. (95% UI, %)	ASMR per 100,000 No. (95% UI)	Death cases No. (95% UI, %)	ASMR per 100,000 No. (95% UI)	Percentage change of death cases No. (95% UI)	EAPC of ASMR No. (95% CI)
East Asia and Pacific	348529.0 (305496.3 to 409729.9,100.0%)	24.71 (21.59 to 29.23)	390192.2 (343689.6 to 440857.4,100.0%)	12.28 (10.83 to 13.83)	11.95% (-11.30% to 37.46%)	-2.23% (-2.29% to -2.18%)
**Sex**						
Male	227630.1 (196525.4 to 270935.1,65.3%)	32.69 (28.16 to 39.11)	268084.1 (231060.4 to 313683.2,68.7%)	17.62 (15.21 to 20.52)	17.77% (-9.47% to 51.41%)	-1.98% (-2.03% to -1.92%)
Female	120899.0 (99050.0 to 144951.5,34.7%)	17.12 (13.96 to 20.73)	122108.2 (103353.3 to 147506.9,31.3%)	7.24 (6.16 to 8.71)	1.00% (-20.94% to 38.59%)	-2.72% (-2.79% to -2.65%)
**SDI***						
High SDI	46276.3 (41740.5 to 48446.8,13.3%)	22.26 (19.94 to 23.47)	39547.9 (34751.1 to 44357.2,10.1%)	7.81 (7.08 to 8.69)	-14.54%	-3.55% (-3.65% to -3.44%)
High-middle SDI	181036.0 (153159.5 to 209723.3,51.9%)	20.44 (17.38 to 23.70)	161435.4 (127496.0 to 197386.0,41.4%)	7.90 (6.26 to 9.60)	-10.83%	-3.29% (-3.42% to -3.16%)

Middle SDI	98851.8 (75877.7 to 135590.4,28.4%)	44.49 (33.35 to 62.88)	160920.7 (129071.7 to 196728.2,41.2%)	32.34 (26.07 to 38.51)	62.79%	-1.00% (-1.07% to -0.93%)
Low-middle SDI	21968.6 (15196.5 to 32423.8,6.3%)	43.15 (29.91 to 65.36)	27473.0 (19479.1 to 36354.4,7.0%)	26.92 (19.29 to 35.54)	25.06%	-1.73% (-1.82% to -1.65%)
Low SDI	396.4 (250.2 to 639.3,0.1%)	14.05 (8.95 to 22.91)	815.2 (601.5 to 1130.0,0.2%)	10.15 (7.42 to 14.09)	105.63%	-1.11% (-1.25% to -0.97%)
**Etiology**						
Hepatitis B	192875.2 (165518.9 to 224064.4,55.3%)	13.54 (11.61 to 15.81)	187957.2 (155533.7 to 221802.3,48.2%)	5.86 (4.87 to 6.89)	-2.55% (-24.27% to 23.06%)	-2.68% (-2.78% to -2.59%)
Hepatitis C	75049.4 (61028.5 to 96734.7,21.5%)	5.44 (4.43 to 7.05)	93263.6 (78477.8 to 109970.3,23.9%)	2.95 (2.48 to 3.47)	24.27% (-4.04% to 53.39%)	-1.99% (-2.02% to -1.97%)
Alcohol use	44810.5 (35495.5 to 56469.1,12.9%)	3.24 (2.58 to 4.09)	65396.9 (54140.4 to 81786.6,16.8%)	1.99 (1.66 to 2.48)	45.94% (14.77% to 75.98%)	-1.44% (-1.51% to -1.38%)
MASLD	8688.1 (5840.3 to 12288.9,2.5%)	0.69 (0.47 to 1.02)	16281.1 (11234.1 to 22427.1,4.2%)	0.51 (0.36 to 0.69)	87.40% (52.65% to 136.68%)	-0.82% (-0.91% to -0.72%)
Other causes	27105.8 (21543.2 to 34326.6,7.8%)	1.79 (1.38 to 2.31)	27293.4 (21060.1 to 35132.0,7.0%)	0.97 (0.77 to 1.22)	0.69% (-19.81% to 25.23%)	-1.83% (-1.88% to -1.77%)
**Country or territory**						

China	179245.6 (151558.1 to 207693.3,51.4%)	20.49 (17.41 to 23.77)	156418.7 (123264.3 to 191699.0,40.1%)	7.69 (6.08 to 9.38)	-12.73% (-35.57% to 16.00%)	-3.07% (-3.18% to -2.95%)
Indonesia	66164.0 (50842.4 to 90324.4,19.0%)	61.16 (45.74 to 86.07)	101589.2 (82643.4 to 122751.7,26.0%)	44.78 (36.49 to 52.51)	53.54% (4.60% to 111.23%)	-0.74% (-0.82% to -0.66%)
Japan	23128.1 (22041.8 to 23697.1,6.6%)	13.78 (13.07 to 14.14)	22386.5 (19414.7 to 24432.8,5.7%)	6.72 (6.15 to 7.13)	-3.21% (-12.61% to 4.05%)	-2.48% (-2.57% to -2.39%)
Philippines	7298.3 (5797.9 to 10184.1,2.1%)	20.69 (15.76 to 30.52)	14513.8 (12059.7 to 17316.5,3.7%)	16.30 (13.58 to 19.31)	98.86% (30.15% to 170.74%)	-0.74% (-0.79% to -0.68%)
Viet Nam	15332.5 (11118.6 to 22748.8,4.4%)	37.90 (27.32 to 57.23)	23875.9 (17902.9 to 30083.2,6.1%)	24.00 (17.98 to 29.93)	55.72% (-12.61% to 140.52%)	-1.51% (-1.54% to -1.48%)
Thailand	9982.9 (8061.8 to 12236.7,2.9%)	25.00 (20.09 to 30.76)	20853.7 (16401.7 to 26461.5,5.3%)	20.46 (16.16 to 25.87)	108.89% (53.33% to 184.29%)	-0.82% (-0.97% to -0.66%)
Myanmar	12021.8 (7699.2 to 18293.6,3.4%)	42.42 (27.48 to 66.04)	13453.4 (9521.8 to 17447.8,3.4%)	24.68 (17.60 to 31.88)	11.91% (-31.30% to 64.32%)	-1.62% (-1.76% to -1.48%)
South Korea	16307.2 (13139.9 to 17620.3,4.7%)	49.82 (41.45 to 53.85)	8993.9 (7926.5 to 11074.2,2.3%)	10.00 (8.82 to 12.31)	-44.85% (-52.67% to -16.40%)	-5.32% (-5.48% to -5.16%)
Malaysia	1741.1 (1560.8 to 1969.9,0.5%)	17.24 (15.46 to 19.50)	4936.0 (4163.2 to 5593.6,1.3%)	17.27 (14.45 to 19.73)	183.50% (123.61% to 239.21%)	0.62% (0.23% to 1.01%)

North Korea	3418.4 (2563.2 to 4778.2,1.0%)	20.27 (15.22 to 28.02)	4811.8 (3243.3 to 6754.5,1.2%)	14.47 (9.90 to 20.20)	40.76% (-8.13% to 110.73%)	-0.99% (-1.03% to -0.94%)
Taiwan (Province of China)	5219.8 (5013.8 to 5428.6,1.5%)	32.65 (31.29 to 33.97)	5776.6 (5227.8 to 6283.7,1.5%)	14.55 (13.25 to 15.76)	10.67% (0.53% to 21.82%)	-2.26% (-2.44% to -2.08%)
Australia	1265.0 (1206.6 to 1328.1,0.4%)	6.65 (6.34 to 6.96)	1999.0 (1823.7 to 2147.3,0.5%)	4.99 (4.60 to 5.34)	58.02% (44.99% to 70.94%)	-1.16% (-1.36% to -0.96%)
Cambodia	4674.6 (3522.8 to 6842.9,1.3%)	88.67 (64.90 to 136.49)	6952.6 (5000.3 to 9097.0,1.8%)	52.93 (38.09 to 69.74)	48.73% (-12.92% to 122.51%)	-1.66% (-1.74% to -1.58%)
Papua New Guinea	250.8 (159.1 to 379.0,0.1%)	10.53 (6.86 to 15.83)	512.3 (392.5 to 663.3,0.1%)	7.66 (5.77 to 9.95)	104.27% (30.11% to 222.09%)	-1.08% (-1.16% to -0.99%)
Laos	1108.6 (790.9 to 1595.5,0.3%)	47.30 (32.65 to 71.32)	1306.4 (937.0 to 1903.0,0.3%)	25.52 (18.40 to 37.20)	17.83% (-14.95% to 64.53%)	-1.85% (-1.97% to -1.73%)
Singapore	210.1 (201.3 to 219.3,0.1%)	8.73 (8.36 to 9.14)	169.3 (153.2 to 181.8,0.0%)	1.99 (1.80 to 2.14)	-19.41% (-26.45% to -12.87%)	-4.40% (-4.51% to -4.28%)
New Zealand	146.1 (137.1 to 153.5,0.0%)	3.84 (3.62 to 4.03)	222.5 (205.0 to 237.5,0.1%)	2.81 (2.61 to 2.99)	52.29% (39.96% to 64.88%)	-1.18% (-1.35% to -1.02%)
Mongolia	660.9 (563.4 to 787.8,0.2%)	57.74 (49.06 to 69.65)	819.1 (687.9 to 964.5,0.2%)	34.98 (29.27 to 41.69)	23.94% (-2.56% to 54.08%)	-0.85% (-1.15% to -0.55%)

Timor-Leste	97.0 (60.9 to 183.0,0.0%)	26.14 (15.65 to 50.24)	182.1 (119.0 to 307.0,0.0%)	20.45 (13.23 to 34.51)	87.73% (31.53% to 179.75%)	-1.05% (-1.17% to -0.93%)
Fiji	53.8 (41.5 to 70.1,0.0%)	11.15 (8.58 to 14.80)	67.8 (49.7 to 88.0,0.0%)	7.99 (5.93 to 10.31)	26.03% (-20.01% to 87.20%)	-0.95% (-1.06% to -0.84%)
Solomon Islands	48.6 (30.2 to 77.2,0.0%)	28.43 (18.72 to 45.05)	120.7 (90.0 to 159.7,0.0%)	27.30 (20.70 to 35.72)	148.38% (54.67% to 287.24%)	-0.08% (-0.14% to -0.03%)
Brunei Darussalam	14.4 (12.0 to 16.8,0.0%)	11.74 (9.76 to 13.86)	29.5 (25.1 to 34.4,0.0%)	7.34 (6.29 to 8.54)	105.19% (65.24% to 153.59%)	-1.45% (-1.59% to -1.30%)
Vanuatu	26.9 (14.8 to 48.1,0.0%)	32.50 (17.62 to 59.44)	54.6 (36.7 to 85.7,0.0%)	24.80 (16.50 to 39.25)	102.80% (40.31% to 193.78%)	-0.94% (-1.00% to -0.89%)
Samoa	16.9 (11.8 to 24.8,0.0%)	17.06 (12.02 to 25.09)	22.7 (16.0 to 30.2,0.0%)	14.22 (10.07 to 18.83)	34.44% (-2.16% to 85.23%)	-0.74% (-0.86% to -0.63%)
Guam	20.7 (18.0 to 24.8,0.0%)	23.54 (20.76 to 27.85)	30.1 (26.1 to 34.2,0.0%)	15.26 (13.21 to 17.30)	45.75% (13.16% to 77.15%)	-1.49% (-1.71% to -1.27%)
Kiribati	14.2 (10.6 to 19.1,0.0%)	30.05 (22.27 to 41.82)	22.7 (16.5 to 30.3,0.0%)	24.90 (18.20 to 32.97)	60.01% (11.68% to 125.09%)	-0.45% (-0.52% to -0.38%)
Micronesia (Federated States of)	17.0 (12.8 to 21.9,0.0%)	28.59 (21.53 to 37.32)	17.3 (11.5 to 23.9,0.0%)	19.51 (13.06 to 26.92)	1.50% (-36.92% to 53.09%)	-1.35% (-1.42% to -1.28%)

Tonga	18.3 (14.4 to 23.7,0.0%)	30.24 (23.54 to 39.25)	18.6 (13.4 to 24.8,0.0%)	22.12 (16.05 to 29.39)	1.44% (-39.23% to 50.89%)	-1.21% (-1.31% to - 1.12%)
Northern Mariana Islands	7.8 (5.9 to 10.3,0.0%)	30.01 (24.29 to 37.71)	11.5 (10.0 to 12.9,0.0%)	21.42 (18.83 to 23.77)	47.93% (8.58% to 99.26%)	-1.31% (-1.46% to - 1.15%)
American Samoa	4.2 (3.3 to 5.1,0.0%)	14.42 (11.54 to 17.82)	6.0 (5.0 to 7.4,0.0%)	11.91 (9.80 to 14.50)	44.83% (6.00% to 97.15%)	-0.70% (-0.83% to - 0.57%)
Marshall Islands	7.1 (5.3 to 9.1,0.0%)	32.62 (23.90 to 43.11)	10.4 (6.5 to 14.9,0.0%)	23.34 (14.76 to 33.23)	47.49% (-21.02% to 124.02%)	-0.91% (-0.97% to - 0.84%)
Palau	2.3 (1.5 to 3.1,0.0%)	19.74 (13.45 to 26.63)	3.5 (2.4 to 4.7,0.0%)	15.00 (10.09 to 19.80)	55.25% (13.07% to 115.36%)	-1.05% (-1.12% to - 0.98%)
Nauru	1.9 (1.2 to 2.5,0.0%)	31.64 (20.05 to 41.05)	1.7 (0.8 to 2.5,0.0%)	22.90 (11.25 to 32.87)	-11.73% (-44.74% to 24.28%)	-0.77% (-0.91% to - 0.63%)
Tuvalu	2.1 (1.6 to 2.8,0.0%)	28.27 (21.14 to 37.12)	2.0 (1.6 to 2.6,0.0%)	18.47 (14.38 to 23.28)	-3.78% (-30.18% to 29.38%)	-1.49% (-1.57% to - 1.40%)

Countries and territories sorted by population in 2021. *: Since there is no predefined SDI region for the East Asia and Pacific (EAP) region, we calculated the Age-Standardized Rate (ASR) for each SDI region by using the population and respective values for each country or territory within the specific SDI region. The cases represent the cumulative sum of the respective values for each country or territory within the specific SDI region

Table S9. Current HCC surveillance recommendations for cirrhotic and non-cirrhotic patients in EAP

Country or territory	Cirrhotic	Non-cirrhotic		Other conditions	Reference
		Chronic hepatitis B	Chronic hepatitis C		
Australia	Any etiologies for all ages	Asian men older than 40 years<br>Asian women older than 50 years<br>People born in sub-Saharan Africa older than 20 years<br>Aboriginal and Torres Strait Islander people older than 50 years			[37]
China	Any etiologies for all ages, especially males aged over 40 years old	Aged 40 years and older	Aged 40 years and older		[38]
Japan	Any etiologies for all ages, especially hepatitis B and C	All ages	All ages		[39]
Malaysia	Hepatitis B, C, alcohol use, genetic hemochromatosis, primary bilary cirrhosis for all ages	Asian males aged 40 years and older<br>Asia women aged 50 years and older		Family history of HCC	[40]
Philippines	Any etiologies for all ages	All ages		Family history of HCC, males aged 40 years and above, and	[41]

				females aged 50 years and above	
South Korea	Any etiologies for all ages	All ages	All ages		[42]
Singapore	Any etiologies for all ages	All ages			[43]
Taiwan	Any etiologies for all ages	All ages	All ages		[44]
Thailand	Any etiologies for all ages	Males aged 40 and above.<br>Females aged 50 and above.<br>Patients with a first-degree relative who has HCC.<br>Patients with advanced firbrosis, assessed by liver biopsy or non-invasive methods.	Patients with advanced fibrosis, assessed by liver biopsy or non-invasive methods, regardless of previous treatment.		[45]

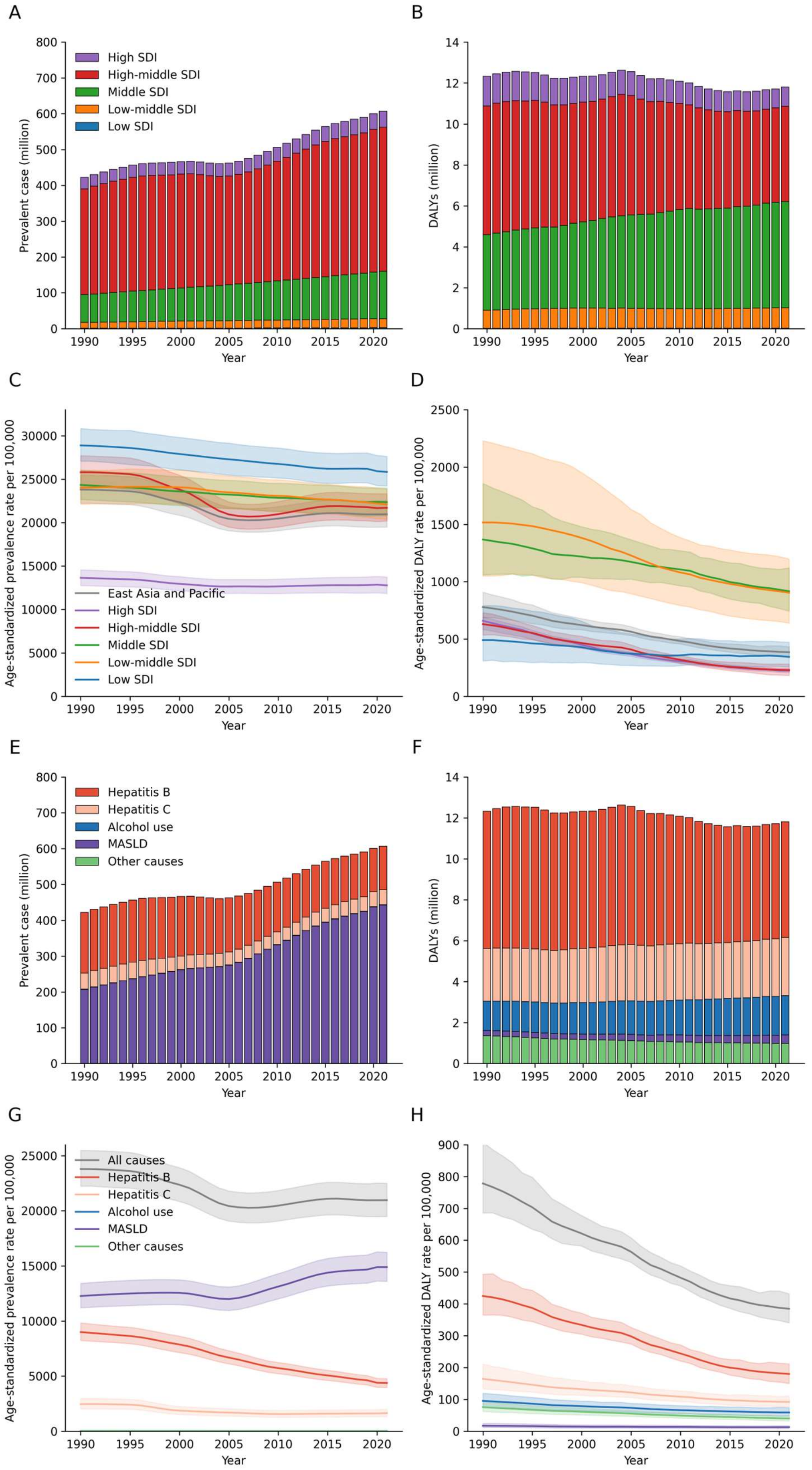

Fig. S1. Changes in prevalence and DALYs of CLD in EAP. (A) Change in prevalent cases by SDI regions. (B) Change in DALYs by SDI regions. (C) Change in age-

standardized prevalence rate in EAP and SDI regions. (D) Change in age-standardized DALY rate in EAP and SDI regions. (E) Change in prevalent cases by etiologies. (F) Change in DALYs by etiologies. (G) Change in age-standardized prevalence rate by etiologies. (H) Change in age-standardized DALY rate by etiologies. Note: All categories, including those with small values such as "low SDI," "alcohol use," and "other causes," are included in the figure using the same plotting method. Due to the relatively low magnitude, these groups may appear visually less prominent.

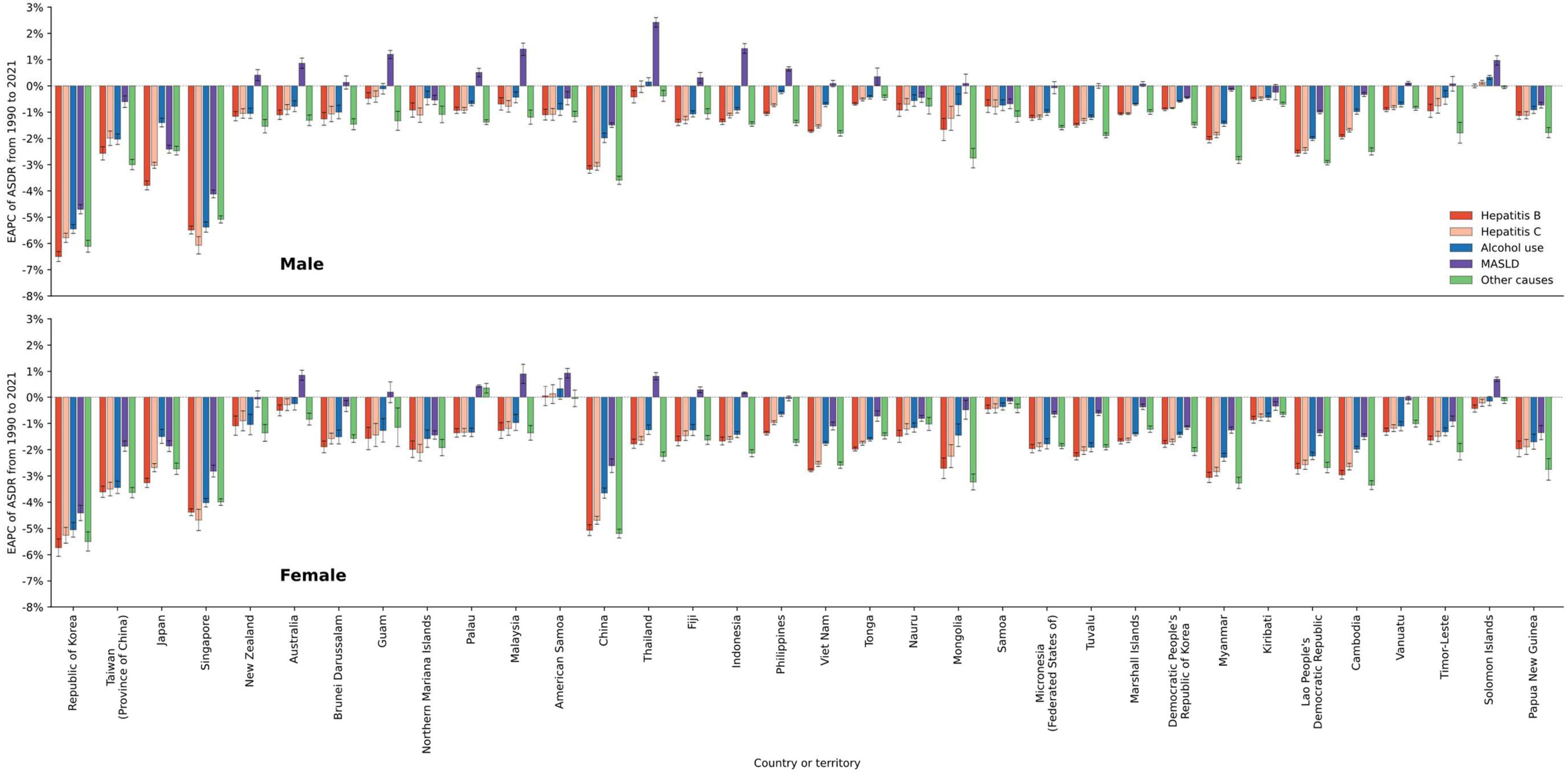

Fig. S2. EAPC of ASDR of CLD stratified by etiologies in EAP, across genders and countries or territories from 1990 to 2021. Countries and territories sorted by SDI in 2021.

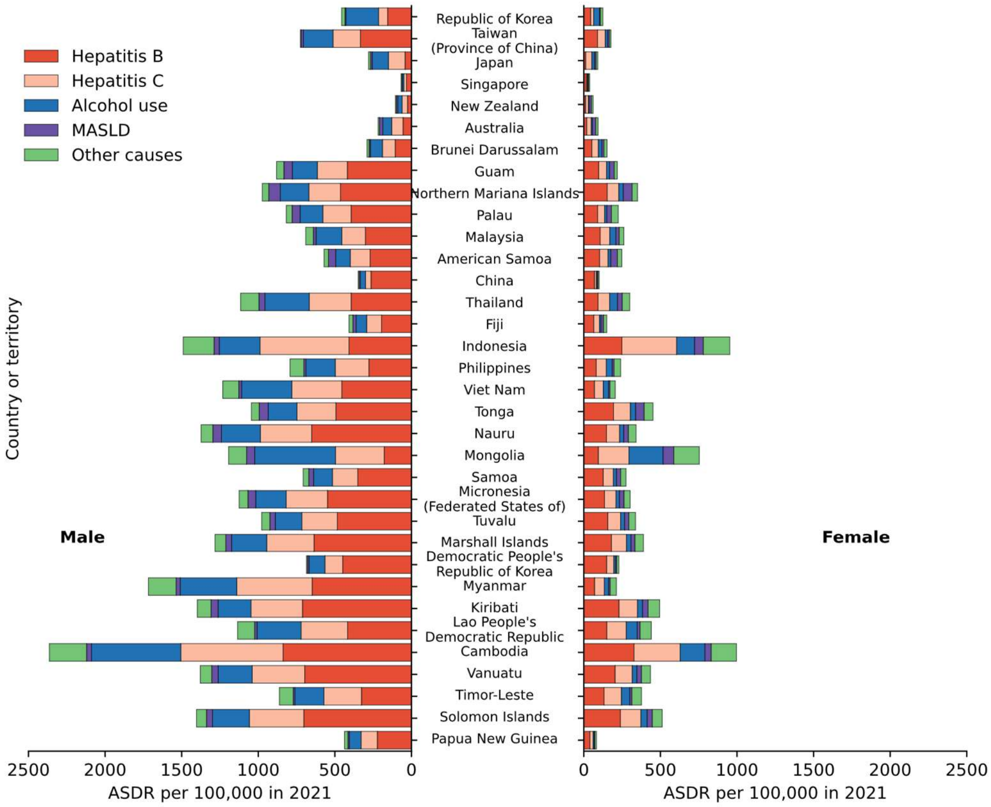

Fig. S3. ASDR of CLD stratified by etiologies in EAP, across genders and countries or territories in 2021. Countries and territories sorted by SDI in 2021.

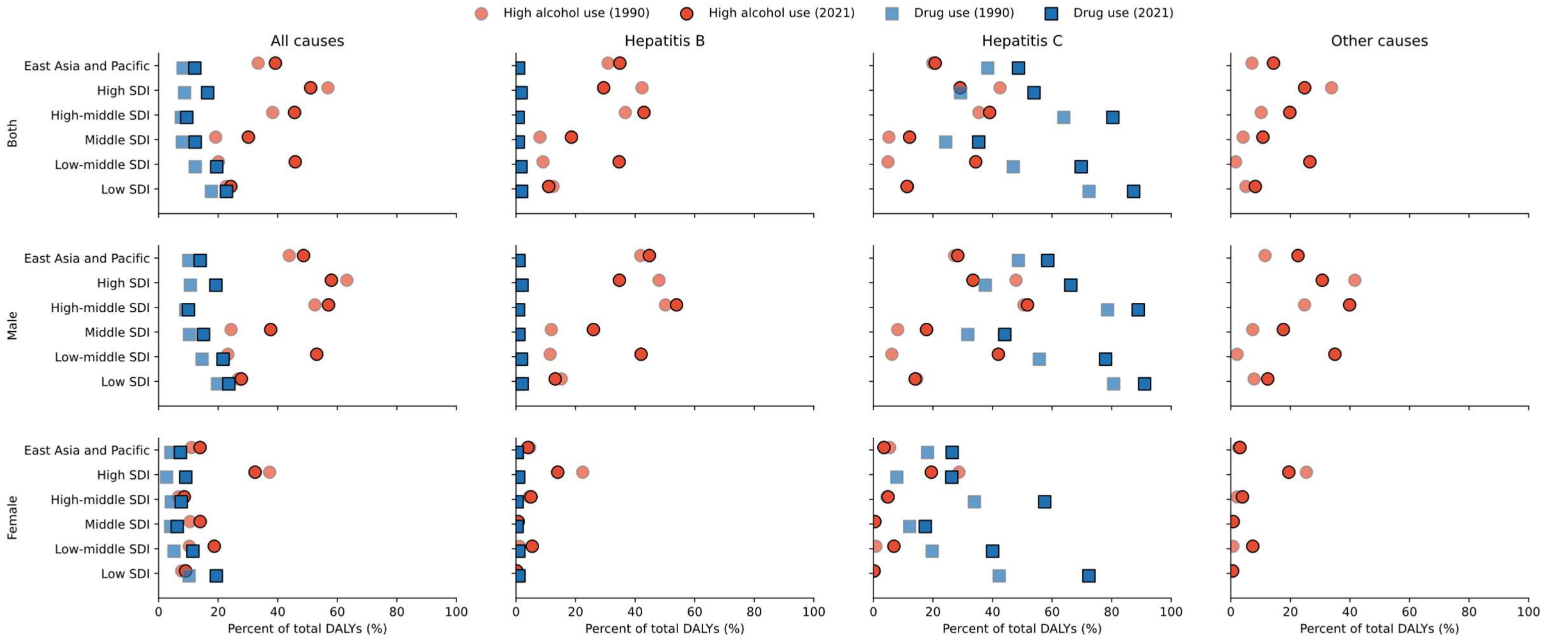

Fig. S4. Proportion of DALYs for CLD by risk factors, stratified by etiology, across genders and countries or territories in EAP in 1990 and 2021.

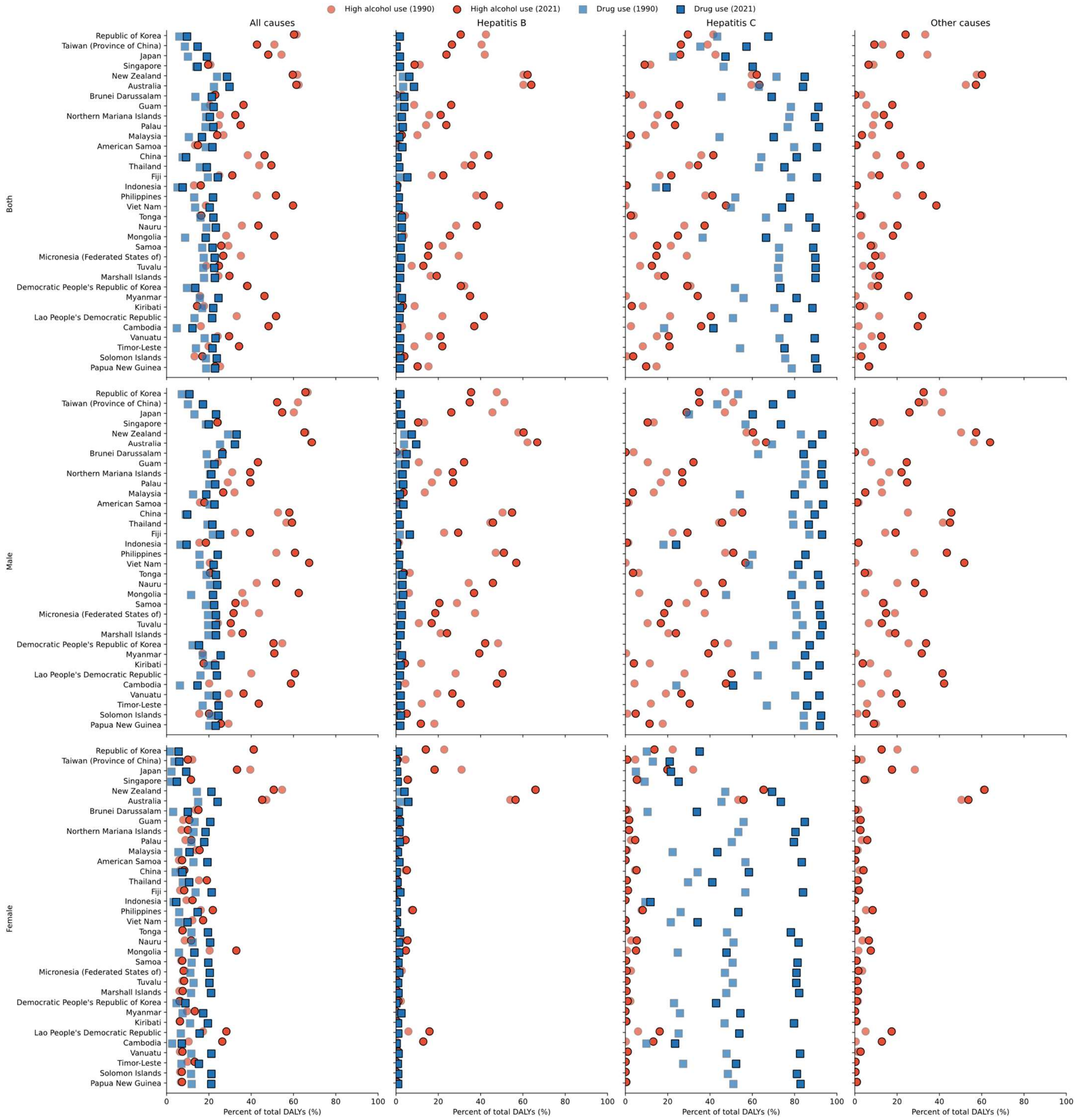

Fig. S5. Proportion of DALYs for CLD by risk factors, stratified by etiology, across genders and countries or territories in EAP in 1990 and 2021. Countries and territories sorted by SDI in 2021.

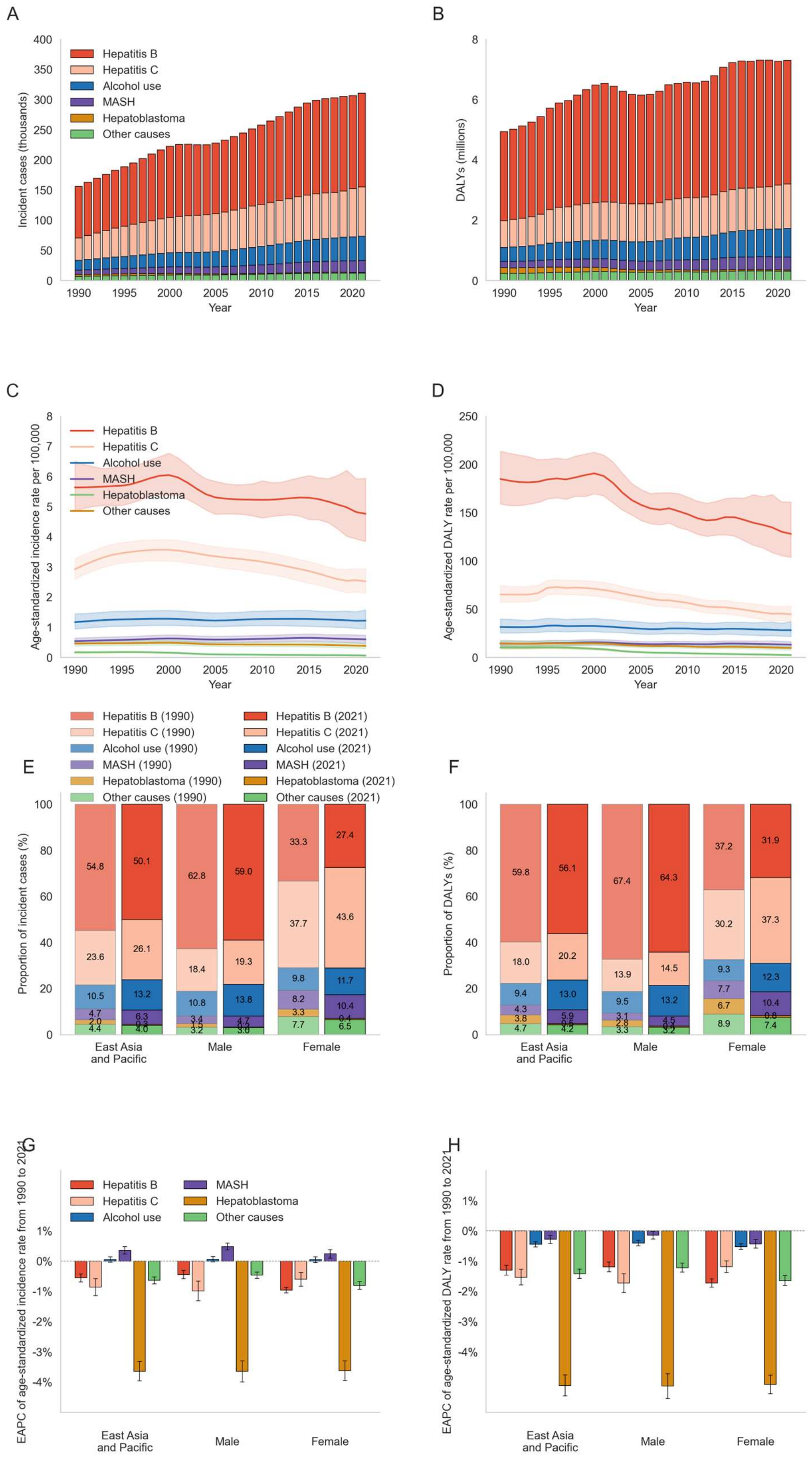

Fig. S6. Changes in incidence and DALYs of liver cancer in EAP. (A) Change in incident cases by SDI regions. (B) Change in DALYs by SDI regions. (C) Change in age-standardized incidence rate in EAP and SDI regions. (D) Change in age-standardized DALY rate in EAP and SDI regions. (E) Proportion of incident cases stratified by etiologies in EAP, across sexes in 1990 and 2021. (F) Proportion of DALYs stratified by etiologies in EAP, across sexes in 1990 and 2021. (G) EAPC of ASIR stratified by etiologies in EAP, across sexes from 1990 to 2021. (G) EAPC of ASDR stratified by etiologies in EAP, across sexes from 1990 to 2021.

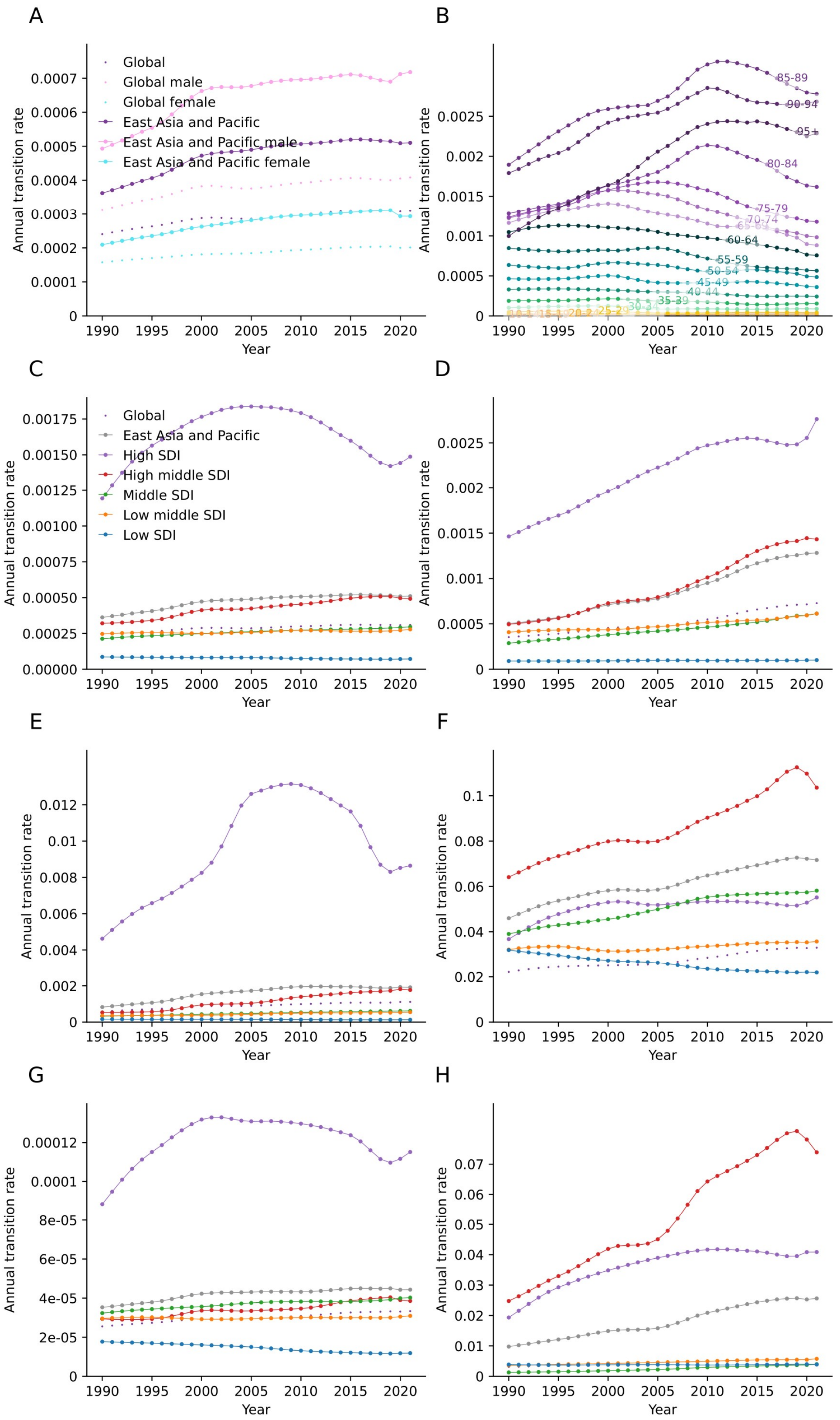

Fig. S7. Annual transition rate from CLD to liver cancer in EAP by sexes, age groups, SDI regions, and etiologies. (A) Annual transition rate globally and in EAP by sexes. (B) Annual transition rate by age groups. (C) Annual transition rate by SDI regions. (D) Annual transition rate from CLD due to hepatitis B to liver

cancer by SDI regions. (E) Annual transition rate from CLD due to hepatitis C to liver cancer by SDI regions. (F) Annual transition rate from CLD due to alcohol use to liver cancer by SDI regions. (G) Annual transition rate from CLD due to MASLD to liver cancer by SDI regions. (H) Annual transition rate from CLD due to other causes to liver cancer by SDI regions. CLD: Cirrhosis and other chronic liver diseases; EAP: East Asia Pacific; SDI: Socio-demographic index; MASLD: Metabolic dysfunction–associated steatotic liver disease.

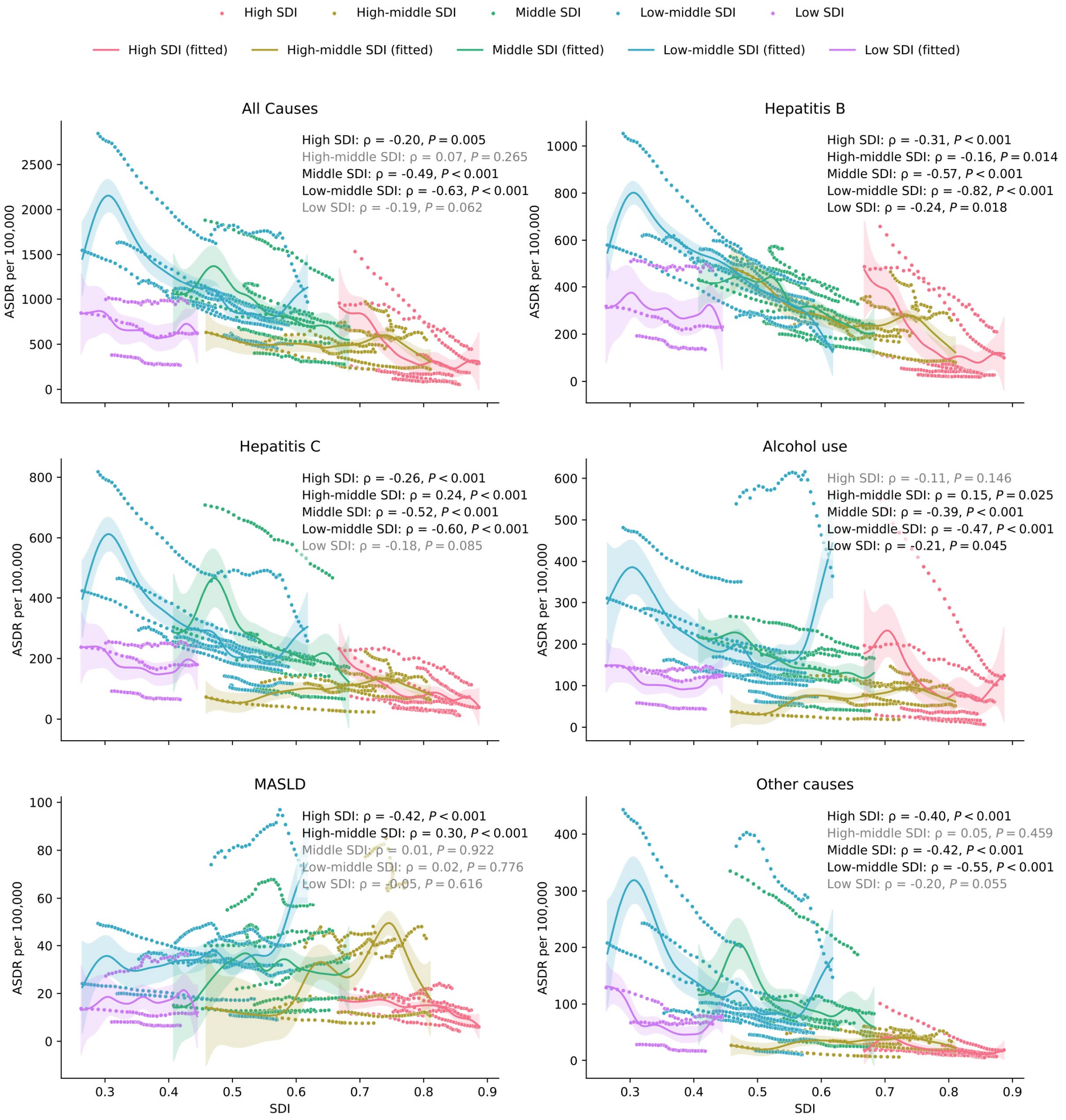

Fig. S8. Relationship between ASDR of CLD and SDI by SDI regions and etiologies in EAP from 1990 to 2021. Spearman correlation analysis was used to assess the strength and direction of the monotonic relationship between SDI and ASDR. Spearman's rank correlation coefficients (ρ) and p-values are shown in

the plot. If the p-value is less than 0.001, it is stated as $P < 0.001$; otherwise, the p-value is reported. $P < 0.05$ were considered statistically significant and are shown in black font, while others are shown in grey. Generalized Additive Models (GAM) were used to fit the relationship using spline functions. The GAM-fitted lines, along with 95% CI, are plotted. ASDR: Age-standardized disability-adjusted life year rate; CLD: Cirrhosis and other chronic liver diseases; SDI: Socio-demographic index; EAP: East Asia Pacific; MASLD: Metabolic dysfunction–associated steatotic liver disease.

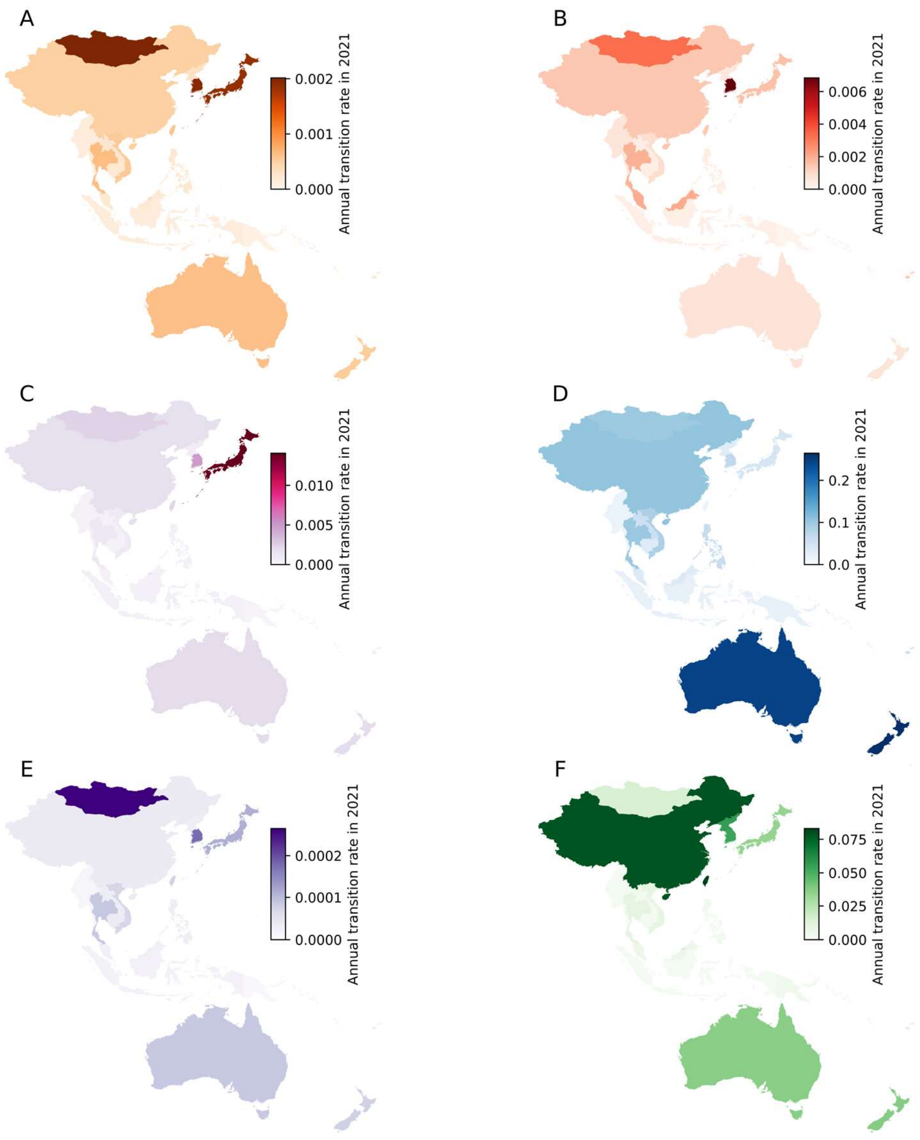

Fig. S9. Annual transition rates from CLD to liver cancer in EAP, stratified by country and etiology, in 2021. (A) Map of annual transition rate from CLD to liver cancer in EAP in 2021. (B) Map of annual transition rate from CLD due to hepatitis B to liver cancer in EAP in 2021. (C) Map of annual

transition rate from CLD due to hepatitis C to liver cancer in EAP in 2021. (D) Map of annual transition rate from CLD due to alcohol use to liver cancer in EAP in 2021. (E) Map of annual transition rate from CLD due to MASLD to liver cancer in EAP in 2021. (F) Map of annual transition rate from CLD due to other causes to liver cancer in EAP in 2021.

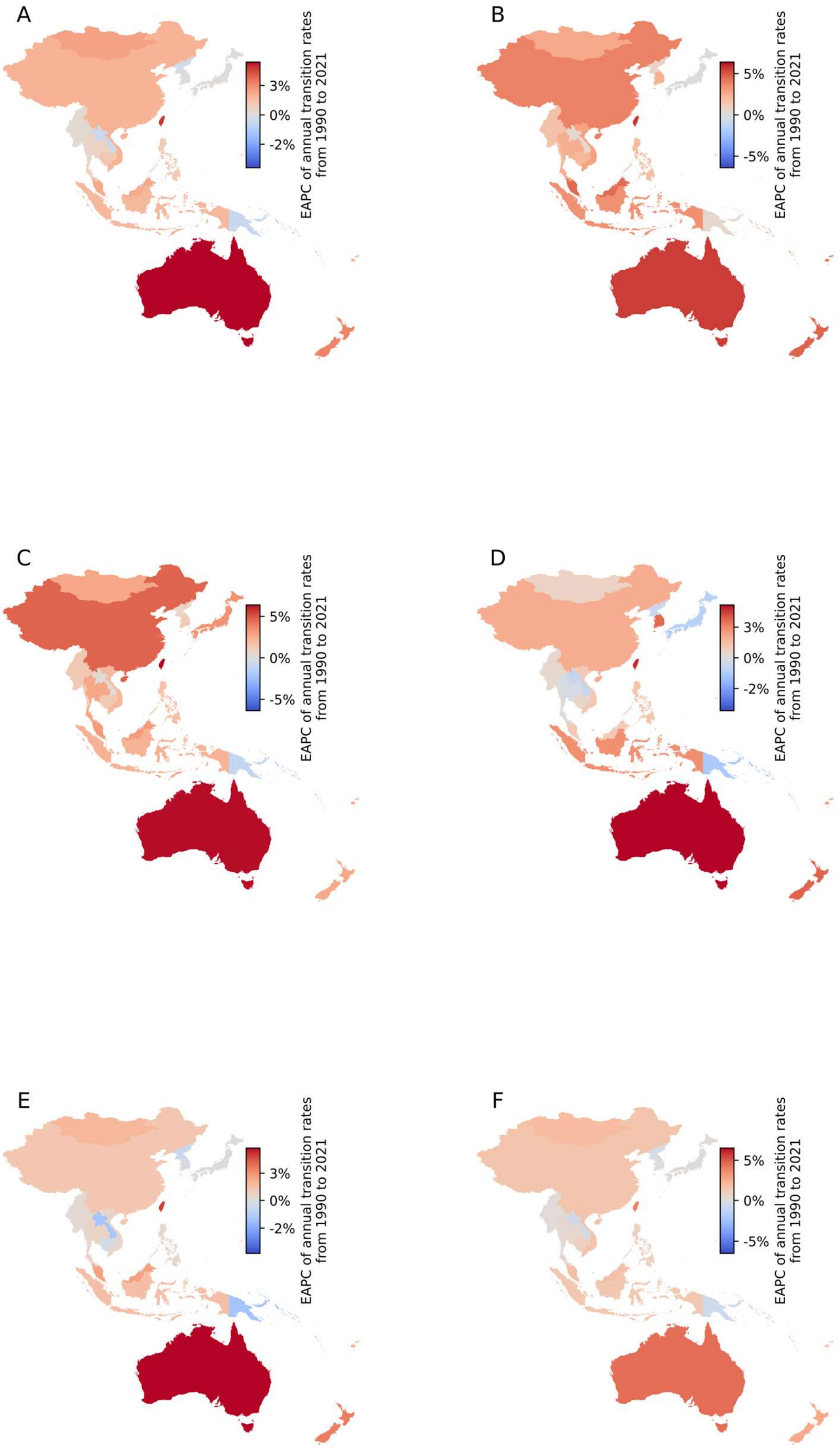

Fig. S10. EAPC of annual transition rates from CLD to liver cancer in EAP, stratified by country and etiology, from 1990 to 2021. (A) Map of EAPC of annual transition rates from CLD to liver cancer in EAP in 2021. (B) Map of EAPC of annual transition rates from CLD due to hepatitis B in EAP in 2021.

(C) Map of EAPC of annual transition rates from CLD due to hepatitis C to liver cancer in EAP in 2021. (D) Map of EAPC of annual transition rates from CLD due to alcohol use to liver cancer in EAP in 2021. (E) Map of EAPC of annual transition rates from CLD due to MASLD to liver cancer in EAP in 2021. (F) Map of EAPC of annual transition rates from CLD due to other causes to liver cancer in EAP in 2021.

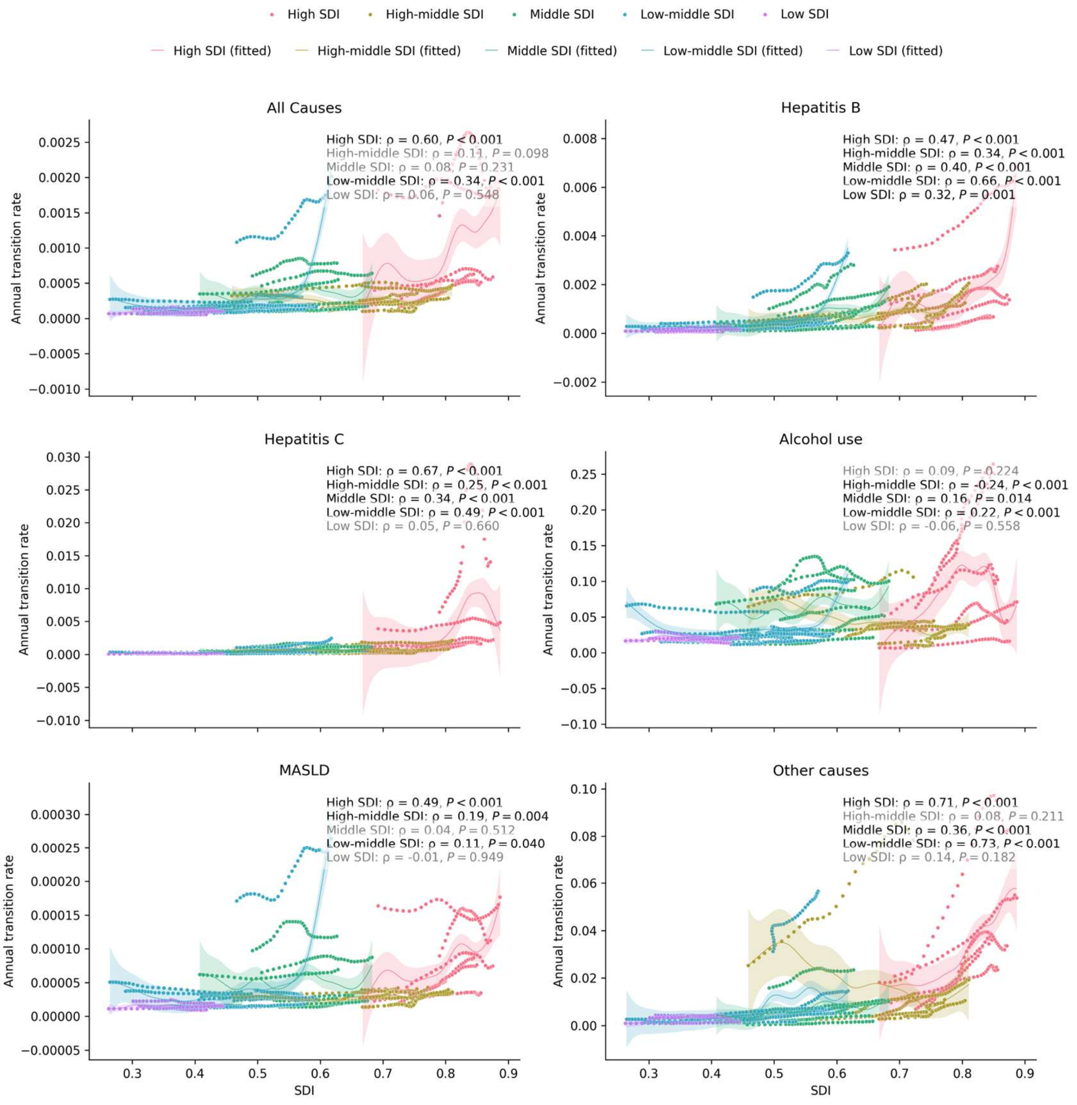

Fig. S11. Relationship between annual transition rates and SDI, stratified by SDI region and etiology, in EAP from 1990 to 2021. Spearman correlation analysis was used to assess the strength and direction of the monotonic relationship between SDI and annual transition rate. Spearman's rank correlation coefficients (ρ) and *P*-values are shown in the plot. If the p-value is less than 0.001, it is stated as $P < 0.001$; otherwise, the p-value is reported. $P < 0.05$ were considered statistically significant and are shown in black font, while

others are shown in grey. Generalized Additive Models were used to fit the relationship using spline functions. The GAM-fitted lines, along with 95% CI, are plotted.

References

1. Baker JL, Collaborators GCoD. Global burden of 288 causes of death and life expectancy decomposition in 204 countries and territories and 811 subnational locations, 1990-2021: a systematic analysis for the Global Burden of Disease Study 2021. Lancet. 2024.
2. IHME. Cirrhosis and other chronic liver diseases. In: Editor(s). Book Cirrhosis and other chronic liver diseases. 2024. https://www.healthdata.org/gbd/methods-appendices-2021/cirrhosis-and-other-chronic-liver-diseases. Accessed Dec 10, 2024.
3. Institute for Health Metrics and Evaluation. 2021 Global Burden of Disease (GBD) study. In: Editor(s). Book 2021 Global Burden of Disease (GBD) study. 2024. https://vizhub.healthdata.org/gbd-results/. Accessed Jul 29, 2024.
4. Ferrari AJ, Santomauro DF, Aali A, et al. Global incidence, prevalence, years lived with disability (YLDs), disability-adjusted life-years (DALYs), and healthy life expectancy (HALE) for 371 diseases and injuries in 204 countries and territories and 811 subnational locations, 1990–2021: a systematic analysis for the Global Burden of Disease Study 2021. The Lancet. 2024.
5. Johnson SC, Cunningham M, Dippenaar IN, et al. Public health utility of cause of death data: applying empirical algorithms to improve data quality. BMC medical informatics and decision making. 2021;21(1):175.
6. Bender RG, Sirota SB, Swetschinski LR, et al. Global, regional, and national incidence and mortality burden of non-COVID-19 lower respiratory infections and aetiologies, 1990–2021: a systematic analysis from the Global Burden of Disease Study 2021. The Lancet Infectious Diseases. 2024.
7. Clegg LX, Hankey BF, Tiwari R, et al. Estimating average annual per cent change in trend analysis. Stat Med. 2009;28(29):3670-82.
8. Liu Z, Jiang Y, Yuan H, et al. The trends in incidence of primary liver cancer caused by specific etiologies: Results from the Global Burden of Disease Study 2016 and implications for liver cancer prevention. J Hepatol. 2019;70(4):674-83.
9. Gupta PD. Standardization and decomposition of rates: A user's manual: US Department of Commerce, Economics and Statistics Administration, Bureau …; 1993.
10. Brauer M, Roth GA, Aravkin AY, et al. Global burden and strength of evidence for 88 risk factors in 204 countries and 811 subnational locations, 1990–2021: a systematic analysis for the Global Burden of Disease Study 2021. The Lancet. 2024;403(10440):2162-203.
11. Sedgwick P. Spearman's rank correlation coefficient. Bmj. 2014;349.
12. Rue H, Martino S, Chopin N. Approximate Bayesian inference for latent Gaussian models by using integrated nested Laplace approximations. Journal of the Royal Statistical Society Series B: Statistical Methodology. 2009;71(2):319-92.
13. Sørbye SH, Rue H. Scaling intrinsic Gaussian Markov random field priors in spatial modelling. Spatial Statistics. 2014;8:39-51.
14. United Nations. World Population Prospects 2024. In: Editor(s). Book World Population Prospects 2024. 2024. https://population.un.org/wpp/Download/Standard/Population/. Accessed Dec 15, 2024.
15. Zhai M, Long J, Liu S, et al. The burden of liver cirrhosis and underlying etiologies: results from the global burden of disease study 2017. Aging (Albany NY). 2021;13(1):279-300.
16. Sepanlou SG, Safiri S, Bisignano C, et al. The global, regional, and national burden of cirrhosis by cause in 195 countries and territories, 1990–2017: a systematic analysis for the Global Burden of Disease Study 2017. The Lancet gastroenterology & hepatology. 2020;5(3):245-66.
17. Sung H, Ferlay J, Siegel RL, et al. Global cancer statistics 2020: GLOBOCAN estimates of incidence and mortality worldwide for 36 cancers in 185 countries. CA: a cancer journal for clinicians.

2021;71(3):209-49.
18. Bruix J, Sherman M, American Association for the Study of Liver D. Management of hepatocellular carcinoma: an update. Hepatology. 2011;53(3):1020-2.
19. Marrero JA, Kulik LM, Sirlin CB, et al. Diagnosis, staging, and management of hepatocellular carcinoma: 2018 practice guidance by the American Association for the Study of Liver Diseases. Hepatology. 2018;68(2):723-50.
20. GUPTA PD. Standardization and decomposition of rates from cross-classified data. Genus. 1994:171-96.
21. Chevan A, Sutherland M. Revisiting Das Gupta: Refinement and extension of standardization and decomposition. Demography. 2009;46(3):429-49.
22. Lin B-Z, Lin T-J, Lin C-L, et al. Differentiation of clinical patterns and survival outcomes of hepatocellular carcinoma on hepatitis B and nonalcoholic fatty liver disease. Journal of the Chinese Medical Association. 2021;84(6):606-13.
23. Jung YB, Yoo JE, Kim KS, et al. Clinical and survival outcomes after hepatectomy in patients with non-alcoholic fatty liver and hepatitis B-related hepatocellular carcinoma. HPB. 2021;23(7):1113-22.
24. Kumar R, Goh B-BG, Kam J-W, et al. Comparisons between non-alcoholic steatohepatitis and alcohol-related hepatocellular carcinoma. Clinical and Molecular Hepatology. 2020;26(2):196.
25. Ahn SY, Kim SB, Song IH. Clinical patterns and outcome of hepatocellular carcinoma in patients with nonalcoholic fatty liver disease. Canadian Journal of Gastroenterology and Hepatology. 2020;2020(1):4873875.
26. Yoon CH, Jin Y-J, Lee JW. Nonalcoholic fatty liver disease-associated hepatocellular carcinoma in a hepatitis B virus-endemic area. European journal of gastroenterology & hepatology. 2018;30(9):1090-6.
27. Kimura T, Kobayashi A, Tanaka N, et al. Clinicopathological characteristics of non-B non-C hepatocellular carcinoma without past hepatitis B virus infection. Hepatology research. 2017;47(5):405-18.
28. Hashimoto E, Taniai M, Kaneda H, et al. Comparison of hepatocellular carcinoma patients with alcoholic liver disease and nonalcoholic steatohepatitis. Alcoholism: Clinical and Experimental Research. 2004;28:164S-8S.
29. Wakai T, Shirai Y, Sakata J, et al. Surgical outcomes for hepatocellular carcinoma in nonalcoholic fatty liver disease. Journal of Gastrointestinal Surgery. 2011;15(8):1450-8.
30. D'Silva M, Na HY, Cho JY, et al. Pathological prognostic factors for post-resection survival in patients with hepatocellular carcinoma associated with non-alcoholic fatty liver disease. Translational Cancer Research. 2021;10(7):3345.
31. Liu M, Tseng T-C, Jun DW, et al. Transition rates to cirrhosis and liver cancer by age, gender, disease and treatment status in Asian chronic hepatitis B patients. Hepatology international. 2021;15:71-81.
32. Yen Y-H, Cheng Y-F, Wang J-H, et al. Characteristics and etiologies of hepatocellular carcinoma in patients without cirrhosis: When East meets West. PLoS One. 2021;16(1):e0244939.
33. Nagaoki Y, Hyogo H, Ando Y, et al. Increasing incidence of non-HBV-and non-HCV-related hepatocellular carcinoma: single-institution 20-year study. BMC gastroenterology. 2021;21(1):306.
34. Shim CW, Park J-W, Kim SH, et al. Noncirrhotic hepatocellular carcinoma: etiology and occult hepatitis B virus infection in a hepatitis B virus-endemic area. Therapeutic Advances in Gastroenterology. 2017;10(7):529-36.
35. Pangilinan P, Raphael JA, Cua IH. Clinical Profile of Patients with Hepatocellular Carcinoma at St. Luke's Medical Center and Cardinal Santos Medical Center from 2003 to 2018. Gut & Liver. 2019;13.
36. Techathuvanan K, Srisajjakul S, Pongpaibul A, et al. Comparison between disease free survival of hepatocellular carcinoma after hepatic resection in chronic hepatitis B patients with or without cirrhosis. J Med Assoc Thai. 2015;98(4):334-42.
37. Lubel JS, Roberts SK, Strasser SI, et al. Australian recommendations for the management of hepatocellular carcinoma: a consensus statement. Medical Journal of Australia. 2021;214(10):475-83.
38. Jie H, Wanqing C, Hongbing S, et al. China guideline for liver cancer screening (2022, Beijing). 临床肝胆病杂志. 2022;38(8):1739-58.
39. Hasegawa K, Takemura N, Yamashita T, et al. Clinical Practice Guidelines for Hepatocellular Carcinoma: The Japan Society of Hepatology 2021 version (5th JSH-HCC Guidelines). Hepatol Res. 2023;53(5):383-90.

40. MINISTRY OF HEALTH MALAYSIA. SERUM ALPHA-FETOPROTEIN (AFP) AND/OR ULTRASOUND (US) FOR HEPATOCELLULAR CARCINOMA (HCC) SCREENING. In: Editor(s). Book SERUM ALPHA-FETOPROTEIN (AFP) AND/OR ULTRASOUND (US) FOR HEPATOCELLULAR CARCINOMA (HCC) SCREENING. 2024. https://www.moh.gov.my/moh/resources/auto%20download%20images/587f12f21cf23.pdf. Accessed Dec 29, 2024.
41. Rizal Medical Center. The Philippine Clinical Practice Guidelines for the Diagnosis and Management of Hepatocellular Carcinoma 2021. In: Editor(s). Book The Philippine Clinical Practice Guidelines for the Diagnosis and Management of Hepatocellular Carcinoma 2021. 2024. https://hsp.org.ph/wp-content/uploads/2021/10/1-The-Philippine-Clinical-Practice-Guidelines-for-the-Diagnosis-and-Management-of-Hepatocellular-Carcinoma-2021.pdf. Accessed Dec 28, 2024.
42. Park J-W. 2022 KLCA-NCC Korea practice guidelines for the management of hepatocellular carcinoma. Clinical and Molecular Hepatology (대한간학회지). 2022;28(4):583-705.
43. Ministry of Health S. Cancer Screening: MOH Clinical Practice Guidelines. In: Editor(s). Book Cancer Screening: MOH Clinical Practice Guidelines. 2024. https://www.hpp.moh.gov.sg/docs/librariesprovider4/guidelines/cpg_cancer-screening.pdf?sfvrsn=7dcee22_0. Accessed Dec 28, 2024.
44. Su TH, Wu CH, Liu TH, et al. Clinical practice guidelines and real-life practice in hepatocellular carcinoma: A Taiwan perspective. Clin Mol Hepatol. 2023;29(2):230-41.
45. Thai Association for the Study of the Liver (THASL). Thailand Guideline for Management of Hepatocellular Carcinoma 2021. In: Editor(s). Book Thailand Guideline for Management of Hepatocellular Carcinoma 2021. 2024. https://thasl.org/wp-content/uploads/2021/04/%E0%B9%81%E0%B8%99%E0%B8%A7%E0%B8%97%E0%B8%B2%E0%B8%87%E0%B8%81%E0%B8%B2%E0%B8%A3%E0%B8%94%E0%B8%B9%E0%B9%81%E0%B8%A5%E0%B8%A1%E0%B8%B0%E0%B9%80%E0%B8%A3%E0%B9%87%E0%B8%87%E0%B8%95%E0%B8%B1%E0%B8%9A%E0%B9%83%E0%B8%99%E0%B8%9B%E0%B8%A3%E0%B8%B0%E0%B9%80%E0%B8%97%E0%B8%A8%E0%B9%84%E0%B8%97%E0%B8%A2-%E0%B8%9E%E0%B8%A8-2564_25-03-64_all.pdf. Accessed Dec 29, 2024.
